# Identifying optimum implementation for human papillomavirus self-sampling in underserved communities: A systematic review

**DOI:** 10.1177/09691413241274312

**Published:** 2024-08-30

**Authors:** Olivia Mackay, Kate Joanna Lifford, Anahat Kalra, Denitza Williams

**Affiliations:** 1 School of Medicine, 2112 Cardiff University, Cardiff, UK; 2 Division of Population Medicine, 2112 Cardiff University, Cardiff, UK

**Keywords:** Human papillomavirus, cervical screening, self-sampling, screening uptake

## Abstract

**Objective:**

To review the existing evidence to identify the optimum methods for implementing human papillomavirus self-sampling to increase screening uptake for underserved groups.

**Setting:**

Specific groups are less likely to participate in cervical screening. These include individuals from low socioeconomic status groups, ethnic minority groups, younger age groups (25–29), older age groups (≥50), with a physical disability, with a learning disability and with an LGBTQ+ identity. The advent of human papillomavirus self-sampling for cervical screening presents an opportunity to promote equitable access to screening. Implementation for human papillomavirus self-sampling can vary, for example, opt-out or opt-in approaches. However, it is unclear which of these is the best method of offering human papillomavirus self-sampling to underserved groups.

**Methods:**

Six databases were searched through May 2023. Studies comparing cervico-vaginal human papillomavirus self-sampling provision using different implementation strategies with the standard screening pathway in underserved groups were identified. A narrative synthesis was conducted.

**Results:**

In total, 4574 studies were identified; 25 studies were included, of which 22 were from high-income countries. Greater uptake was found for offering human papillomavirus self-sampling compared to standard clinician-based sampling. Directly mailing human papillomavirus self-sampling kits to participants resulted in higher uptake of screening than using an ‘opt-in’ approach or standard recall in low socioeconomic status and ethnic minority groups, and older women. Strategies that used community health workers or educational materials increased uptake in ethnic minority and low socioeconomic status groups.

**Conclusions:**

Directly mailing human papillomavirus self-sampling kits to low socioeconomic status groups, ethnic minority groups and older women has the potential to increase uptake of human papillomavirus self-sampling. Using community health workers to offer human papillomavirus self-sampling should be considered for ethnic minority and low socioeconomic status groups. Further research exploring the preferences of younger women is needed.

## Introduction

Cervical cancer (CC) is the second most diagnosed cancer in women under 45 years old.^1^ High-risk oncogenic subtypes of human papillomavirus (HPV16, HPV18) cause approximately 70% of CC cases.^2,3^ As a result of the link between HPV and CC, focus has turned to prevention of persistent HPV infection.^4^ The UK has a successful HPV vaccination programme that is offered to 12–13-year-old girls (since 2008) and boys (since 2019).^5^ The vaccination programme has been highly effective at reducing the incidence of pre-cancerous changes and CC.^6^ This complements the National Health Service (NHS) cervical screening programmes (CSPs) which are offered at routine intervals to women between the ages of 25 and 64.^7–10^ As of 2024, all UK CSPs replaced cytology-based screening with primary HPV testing.^7^ In England, cervical screening currently prevents 70% of CC deaths; however if all eligible individuals were engaged with regular screening, it is estimated that 83% of deaths could be prevented.^11^ The introduction of vaginal self-sampling for cervical screening presents a new opportunity to increase screening uptake in the UK by promoting choice in sample collection method.^12,13^ However, although choice is generally seen as a positive step in patient activation, it is critical to understand the optimum way to implement self-sampling for all eligible individuals to ensure equitable access to screening choices.

High uptake of screening is associated with lower CC incidence and mortality,^11,14^ but participation in cervical screening in the UK has been steadily declining with coverage decreasing from 75.7% in 2011 to 70.2% in 2021.^15^ There are a range of barriers that prevent uptake of cervical screening such as embarrassment and fear of the procedure, inconvenient appointment times, language barriers and gender-discordant sample takers.^16,17^

Specific groups of the population have been identified as less likely to engage with cervical screening than the rest of the population; these groups are commonly referred to as ‘underserved’ groups. These underserved groups have been identified as women from ethnic minority groups,^18,19^ members of the LGBTQ+ community,^20^ lower socioeconomic status (SES) groups,^21^ younger women (aged 25–29),^22,23^ older women (≥50)^23^ and those with a learning disability^24^ or physical disability.^25^

Human papillomavirus self-sampling (HPVSS) has been identified as a method to overcome some of the barriers associated with clinician-based screening, thereby increasing uptake in non-attenders and reducing screening inequalities.^26^ With self-sampling, the individual receives a sampling kit and takes their own cervico-vaginal sample. The sensitivity of these self-collected samples is comparable to that of clinician-collected samples.^12,27^ Self-sampling has been shown to be an acceptable method of screening to individuals represented in research^26,28^ and to increase screening uptake among underserved groups.^29^ One UK study found offering non-attenders self-sampling generated a 2.25-fold higher participation rate than clinician-based sampling.^30^

There are various implementation strategies for HPVSS. Those eligible for screening can be offered the option of ordering their own kits through a website, letter, email or telephone service, or by being given the kit at their general practitioner surgery or clinic.^31^ These strategies are broadly known as the ‘opt-in’ approach – where women request their self-sampling kit. The other main strategy is the ‘opt-out’ approach, where self-sampling kits are mailed directly to all who are eligible, without participants having to request a kit.^31^ There are benefits with the latter approach, because non-attenders are more likely to engage with screening, however it is a costly method and wastes unused kits.^32^

This review builds on a recent meta-analysis, which found that directly mailed kits increased screening participation among non-attenders.^29^ However, Yeh et al. did not examine the best ways of implementing HPVSS among underserved groups.^29^ Different implementation approaches might be needed for different groups. The European Cancer Organisation has recommended that ‘self-sampling forms a central component of national cervical cancer screening programmes’.^33^ Several countries, such as Australia (offering HPVSS in primary care) and Denmark, have already implemented self-sampling as part of their CSPs.^34^ The UK plans to follow suit, with preliminary results from UK feasibility trials showing HPVSS can increase screening in non-attenders.^35^ CSPs across the UK nations are in discussion about the possibility of implementing HPV self-sampling within their programmes. The aims of this review were (a) to identify the optimum implementation strategy(s) for HPVSS to increase uptake in different underserved groups, and (b) to understand the impact of HPVSS implementation strategies on acceptability of HPVSS in different underserved groups.

## Methods

The review was registered on PROSPERO (CRD42023390276) and reported in line with the Preferred Reporting Items for Systematic Reviews and Meta-analyses (PRISMA) guidelines.^36^

### Definitions

HPVSS was defined as the method of an individual collecting their own cervico-vaginal HPV sample (without use of a speculum).^37^ This review did not include HPV urine sampling.

Underserved communities were defined as groups who have been identified as less likely to participate in cervical screening. These included those from low-SES groups, ethnic minority groups, younger women (≤29 years), older women (≥50 years), those with an LGBTQ+ identity, those with physical disabilities and those with learning disabilities.^19^

The term ‘women’ has been used throughout this review to refer to all individuals who are eligible for cervical screening (i.e. people with a cervix). We note that this may include transmen and non-binary individuals.

### Search strategy

Medline, Embase, Scopus, PsycINFO, Web of Science and CINAHL were searched through January 2023 using a combination of search terms for HPV, self-sampling and each underserved group. The search was updated in May 2023 and articles with publication date of 2023 were reviewed. The full search strategy is included in Appendix A.

### Study eligibility

The inclusion and exclusion criteria are summarised in Table 1. No country or publication date restrictions were applied. Studies were not limited by design. Only studies that included a sub-group of participants from an underserved group identified above were included.

**Table 1.** Inclusion and exclusion criteria.

Inclusion	Exclusion
Participants: part of an underserved group Intervention: implementation of HPVSS using cervico-vaginal samples Comparison: alternative HPVSS implementation strategy or control (e.g. Pap smear, standard recall, clinician-based sampling) Outcomes: uptake (percentage or proportion that completed screening) or acceptability (e.g. attitudes, perceptions, preferences, willingness to repeat, pain, satisfaction)	Not primary research Intervention: anal or urine samples Intervention: hypothetical scenarios (e.g. vignettes, or prior experience not part of the study) Not available in the English language No full text available Conference abstract

HPVSS: human papillomavirus self-sampling.

### Data extraction and synthesis

Initial title and abstract screening was performed to identify relevant articles (OM). Articles were then screened at full text (OM). Twenty per cent of all articles were independently screened by a second reviewer (AK) at each stage. Discrepancies were resolved through discussion. Endnote reference managing software was used to manage exported papers.

Data extraction was completed by one reviewer (OM) using a piloted data extraction sheet (Appendix B) which included: authors, year, country, study design, study population, aims, screening interventions, outcomes measured and main findings. Due to the heterogeneity of the data, a narrative approach was used to synthesise the evidence. The synthesis was conducted using four stages of narrative synthesis: preliminary synthesis of studies, exploration of relationships in the data, theory development and the assessing robustness of the synthesis.^38^

### Assessing bias

One reviewer assessed the quality of the studies. The Cochrane Risk-of-Bias (RoB-2) tool was used to assess risk of bias in the randomised studies.^39^ The Risk Of Bias In Non-randomised Studies – of Interventions (ROBINS-I) tool was used to assess risk of bias in the observational studies.^40,41^

## Results

### Study selection

**Figure 1.**
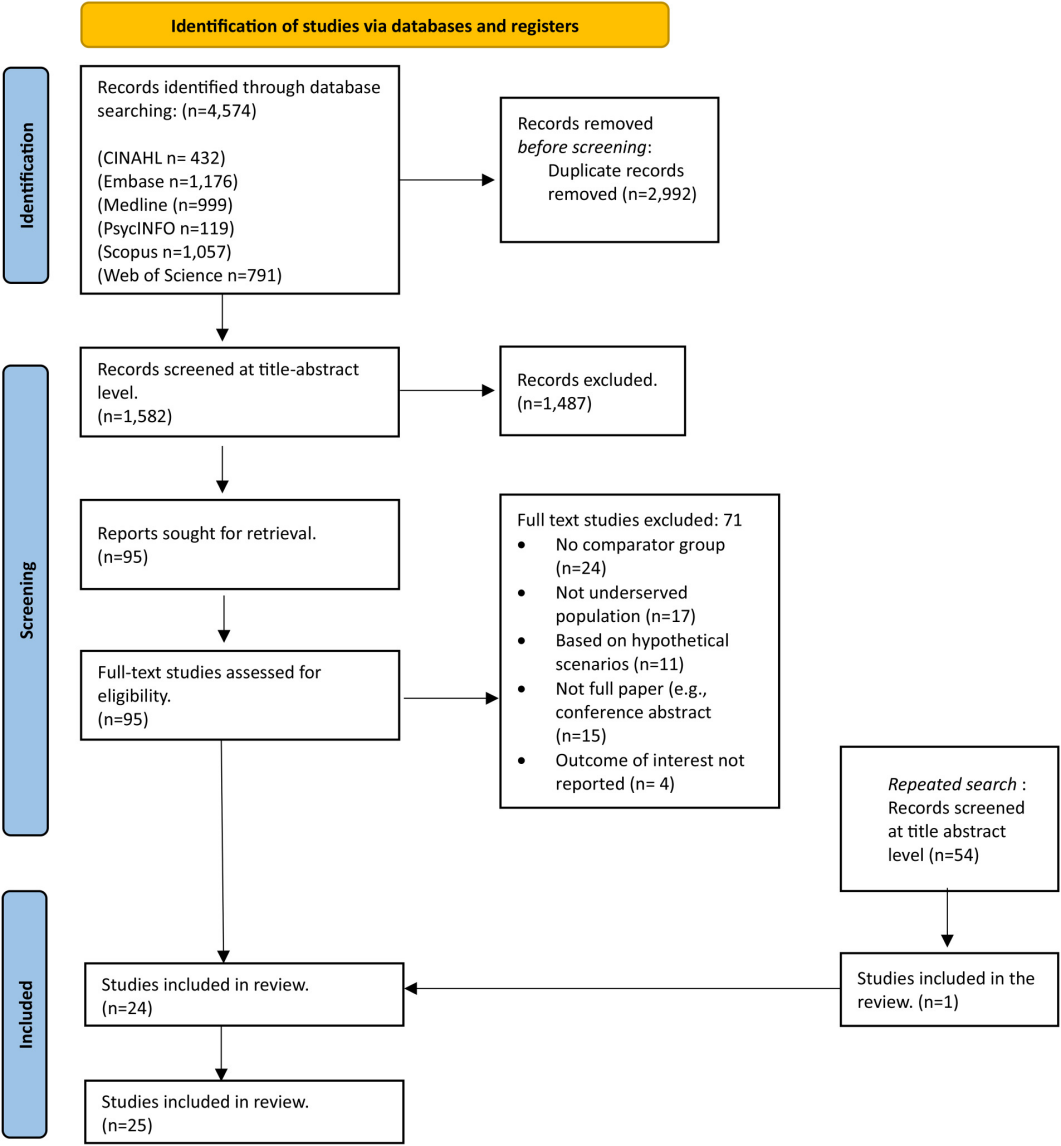
PRISMA flow diagram of study selection process.

Database searching retrieved 4574 articles (Figure 1). After de-duplication, 1582 citations were screened at the title-abstract level. Ninety-five full-text articles were screened; 24 articles were identified as meeting the eligibility criteria and 71 articles were excluded (reasons listed in Figure 1 and Appendix C). From the repeated search, 54 articles were screened and one relevant article was identified. Thus 25 articles were included in the review.

### Study characteristics

The study characteristics of the 25 studies are shown in Table 2. The studies have been grouped by underserved group. Fifteen of the studies were randomised controlled trials (RCTs) and nine were observational (four prospective cohort and five cross-sectional) studies. One study combined the data from two RCTs,^42^ so the two original studies were appraised for their risk of bias.^43,44^ Articles were published between 2005 and 2023. There was a total of 132,039 participants aged between 25 and 70 across the studies. Sample sizes of the included studies ranges from 63 to 25,061. The included studies came from 13 countries (eight from the USA,^45–52^ four from the UK,^53–56^ two from New Zealand,^57,58^ two from Finland^59,60^ and one from each of the following countries: Australia,^61^ Belgium,^62^ Denmark,^63^ France,^64^ Hong Kong,^65^ India,^66^ Jamaica,^67^ Mexico^68^ and the Netherlands^42^). Twenty-two studies were conducted in high-income countries. Nineteen studies only included ‘non-attenders’. The definition used to classify ‘non-attenders’ varied by study, therefore individual definitions have been included for each study (Table 2). No studies reported on people with an LGBTQ+ identity, physical disabilities or learning disabilities, and only one reported on younger women.^56^ Comparators have been listed in Table 2 as study authors reported them (e.g. Pap smear, cytology-based). These screening methods are near identical in procedure; therefore the term clinician-based sampling has been used to refer to them in the synthesis.

**Table 2.** Summary characteristics of included studies: comparing cervico-vaginal human papillomavirus self-sampling (HPVSS) provision using different implementation strategies with the standard screening pathway in underserved groups.

Author, year, country	Study design and aim	Participants	Intervention, comparator and device	Outcomes	Main findings	Notes
**Low socioeconomic status (SES) groups**						
Anand et al., 2022,^66^ India	Design: Cross-sectional Aim: To assess if the modality of communication may influence cervical cancer (CC) screening uptake and quality of self-sampling.	*n* = 500 Women aged 30–55 Low-income	In-person health education session vs written pamphlet education before HPVSS Device: not reported	Uptake at 3 days Acceptability	Uptake in in-person health education arm 245/250 (98%) vs written pamphlet education: 205/250 (82%). The acceptance of HPVSS was 100% (250) in the in-person health education arm and 94% (235/250) in the written pamphlet education arm.	Women returned their kits to a community health worker (CHW). No statistical significance reported for any outcomes.
Anhang et al., 2005,^45^ USA	Design: Cross-sectional questionnaire Aim: To determine the acceptability of self-collection of specimens for HPV DNA testing in US clinic settings.	*n* = 172 Women aged 25–65 Low-income	HPVSS in clinic then clinician-based sampling Device: Dacron swab	Acceptability Perceived efficacy Discomfort Preference	Those who preferred self-sampling: Participants cited ease of use (69%), less painful procedure (62%), ‘could do it myself’ (56%) and privacy (52%) as desirable characteristics of self- sampling. 21% believed that self- collection of specimens was better at finding cancer. 31% were not sure whether they had performed the self-collection correctly, 14% thought it was not good at detecting cancer. 7% found it painful or physically uncomfortable. Of those expressing a preference, 32% preferred the self-collected test, 68% preferred clinicians. Women who are not Hispanic were more likely to prefer self-collection (28.3% vs 48.6%, *p* < 0.05).	Women self-presented in free healthcare clinic, identified as low-income. Women given educational talk then self-sampling kit to use in clinic bathroom. Then underwent clinician sampling. Unclear how acceptability was measured. No statistical significance reported for questionnaire, statistical analysis done for differences between sub-groups. Sub analysis by ethnicity for preference.
Kilfoyle et al., 2018,^46^ USA	Design: Cross-sectional questionnaire Aim: To assess attitudes, experiences and preferences regarding HPV testing by home-based self-collection with Pap testing among low income under screened women	*n* = 100 Women aged 30–65 Low-income Non-attenders (no Pap test in the last 4 years)	Directly mailed HPVSS kit vs Pap smear test HPVSS Device: Brush by Rovers Medical Devices	Attitudes Willingness to repeat Perceived safety Discomfort Convenience Preference	Positive thoughts about the test: HPVSS 81%, Pap test 75% (*p* = 0.353). 98% would repeat HPVSS and the Pap test (98%) (*p* = 1.00). 99% agreed HPVSS was safe vs 97% agreed Pap test were safe (*p* = 0.317). More women reported experiencing ‘a little physical discomfort’ (Pap 43% vs self 18%, *p* < 0.001), ‘a lot of physical discomfort’ (5% vs 0%, *p* < 0.001) or ‘a little pain’ (30% vs 10%) (*p* = 0.001) from the Pap test. 13% agreed it was hard to find the time to perform HPVSS, 31% agreed it was hard to find the time to attend Pap smear test (*p* = 0.003). 51% of women stated that they would prefer an HPV self-test, 19% stated they would prefer the Pap test, and 27% had no preference (*p* < 0.001 Pap vs self). Preference sub-analysis by ethnicity: 61% of White participants and 45% of Black participants preferred HPVSS (odds ratio (OR) 0.52, 95% confidence interval (CI) 0.3 to 0.92).	Defined low income as (a) had children that qualified for the federal school lunch programme, (b) had Medicaid or Medicare Part B insurance or (c) were uninsured and living at or less than 200% of the federal poverty level (determined by household income and size). Pre-paid return envelope. Incentives given for completion. This study performed a sub analysis for ethnicity.
Lazcano et al., 2011,^68^ Mexico	Design: RCT Aim: To assess relative detection rates, relative sensitivity (ratio of the relative detection rates), and positive predictive value for CIN 1–3 and invasive CC in the HPV group vs the cytology group	*n* = 25,061 Women aged 25–65 Low income	Home-based HPVSS offered by community nurses (CNs) vs written invitation for cytology at local health centre. HPVSS Device: Digene conical-shaped brush	Uptake	Home-based HPVSS 98% (9202/9371), invitation for cytology 87% (11,054/12,731) (*p* = 0.001).	Low-income rural areas. CNs used to recruit women face-to-face and give instructions on HPVSS. Women returned samples immediately to CNs. Clinician-based sampling was cytology at local health centre Follow-up not clearly reported.
McFarlane et al., 2022,^67^ Jamaica	Design: Cohort Aim: To determine the efficacy of culturally targeted fear appeal messages to increase screening uptake	*n* = 163 Women aged 30–65 Low-income Non-attenders (no Pap test in last 3 years)	HPVSS kit with culturally tailored message vs HPVSS kit without culturally tailed message (plain kit) Device: Cotton swab	Acceptability Perceived efficacy Perceived threat Attitudes	Acceptability between groups (*F*_(1,147) _= 2.97, *p* = 0.09). Perceived efficacy (*F*_(1,148) _= 0.12, *p* = 0.73). Perceived threat (*F*_(1,148) _= 3.65, *p* = 0.06). Kit attitudes (*F*_(1,147) _= 8.00, *p* = 0.01). The Plain kit had more positive kit attitudes (mean = 6.24) than the culturally targeted (mean = 5.86).	Women received an educational session before the kit. The culturally tailored kit included illustrations, ‘vibrant Jamaican colours’ and diagram explaining cancer progression. Control was plain kit. Monetary incentive for completion.
Pretsch et al., 2023,^47^ USA	Design: RCT Aim: To identify whether mailing HPV self-collection kits to women's homes in conjunction with providing appointment scheduling assistance resulted in increased uptake of CC screening compared with offering scheduling assistance alone.	*N* = 697 Women aged 25–64 Low-income Non-attenders (no self-report of having Pap test within 4 years)	Directly mailed HPVSS kit and assistance in scheduling in-clinic appointments vs scheduling assistance (control) Device: Viba brush (Rovers Medical Devices)	Uptake at 6 months	Directly mailed HPVSS kit and assistance in scheduling in-clinic appointments (317/438, 72%) vs scheduling assistance (control) (85/438, 37%). Risk ratio 1.93 (95% CI 1.62 to 2.31).	Low-income defined as 250% or less of the US Federal Poverty Level. Uninsured or enrolled in Medicaid or Medicare. Women given reminder letter after 3 weeks and phone call after further 2 weeks. Used ‘intensive community outreach campaigns’ to reach women. Uptake was defined as attending a screening appointment at any clinic or testing negative for high-risk HPV on self-collected samples.
Sancho et al., 2013,^64^ France	Design: RCT Aim: To compare rates of participation between HPVSS and Pap smear	*n* = 18,730 Women aged 35–69 Low-income Non-attenders (had no Pap smear test for more than 2 years)	Direct mailing of HPVSS kit vs standard second reminder letter for Pap smear Device: Dacron swab	Uptake	Directly mailed HPVSS (18.3%) vs Pap smear (2%) (*p* ≤ 0.001).	Participants identified through a system that provides free healthcare to the poorest people in the area. Both Pap smear test and HPVSS were free. Women were sent a letter before being given a SS kit. Follow-up period not clearly recorded.
Tranberg et al., 2018,^63^ Denmark	Design: RCT Aim: To assess if offering HPVSS kits has an effect on screening participation among various socioeconomic groups	*n* = 3061 sub-group with low income (*n* = 9791 overall sample from various socioeconomic groups) Women aged 30–64 Non-attenders (due their second reminders)	Direct mailing of self-sampling kit vs opt-in vs standard second reminder for clinician-based screening Device: Evalyn brush	Uptake at 6 months	Directly mailed (31.4%) vs opt-in (24.7%) vs reminder for clinician-based screening (19.5%). Directly mailed vs reminder participation difference = 11.9 (95% CI 8.2 to 15.6). Opt-in vs reminder participation difference = 5.2 (95% CI 1.6 to 8.8). Directly mailed vs opt-in participation difference = 6.7 (95% CI 2.8 to 10.6).	Study conducted as part of national screening programme. Participants classified by SES.

**Table:** 

**Ethnic minority groups**						
Brewer et al., 2021,^57^ New Zealand	Design: RCT Aim: To assess whether two specific invitation methods for self-sampling improved screening participation over usual care among the least medically served population	*n* = 3553 Māori, Pacific and Asian women aged 30–69 Non-attenders (not screened in the last 5 years)	Directly mailed HPVSS vs HPVSS offered in clinic vs standard written invitation for cytology (usual care) HPVSS Device: FLOQSwab™	Uptake at 3 months	Directly mailed HPVSS 14% (205/1467), *p* < 0.0001 compared to usual care. In-clinic HPVSS 6.4% (100/1574) (*p* = 0.002 compared to usual care). Usual care = 2.7% (14/512). Sub-group analysis for SES: New Zealand deprivation quintile 5: directly mailed HPVSS (8.7%, *p* = 0.015 compared to usual care), in-clinic HPVSS (3.9%, *p* = 0.604), usual care (3%).	Sub-analysis for lowest deprivation quintile ethnic minority group.
Carrasquillo et al., 2018,^48^ USA	Design: RCT Aim: To compare a CHW-led HPVSS intervention with standard CC screening approaches.	*n* = 601 Black, Haitian and Hispanic women aged 30–65 Non-attenders (no Pap smear in the last 3 years)	Culturally tailored public health outreach (Outreach) vs CHW navigation to local facilities for Pap smear (Navigation) vs CHW-facilitated HPVSS (HPVSS) Device: Not reported	Uptake at 6 months	31.3% (57/182) Outreach, 42.5% (90/212) Navigation and 77.3% (160/207) for HPVSS option. HPVSS 77.3% vs Outreach 31.3% (OR 7.47, *p* < 0.01). HPVSS 77.3% vs Navigation 42.5% (OR 4.61, *p* < 0.01).	Women recruited from three ‘ethnic neighbourhoods’ in South Florida. Women in the HPVSS group were given the choice between Pap smear and HPVSS. HPVSS was supported by CHW. The HPVSS and navigation group received one-to-one education session from CHW.
Castle et al., 2011,^49^ USA	Design: Feasibility study Cross-sectional Aim: To determine what type of free screening intervention women living in Mississippi Delta chose	*n* = 119 African-American women aged 25–65 Non-attenders (no Pap smear in the last 3 years)	HPVSS vs Voucher for Pap smear Device: Not reported	Uptake	77/119 (64.7%) chose HPVSS and 42/119 (35.3%) chose clinic-based Pap testing. 17/42 (40.47%) completed Pap smear; 62/77 (80.52%) completed HPVSS (*p* = 0.0001).	Door-to-door recruitment with educational session about options. Women got to choose either HPVSS or Pap. Women could return HPVSS immediately to study personnel or return the kits by mail. Follow-up not clearly recorded.
Kobetz et al., 2018,^50^ USA	Design: RCT Aim: To compare two modes of self-sampling delivery: SS mail or in-person SS	*n* = 600 Ethnic minority (Hispanic, Haitian, non-Hispanic Black) women aged 30–65 Non-attenders (No Pap smear in the last 3 years)	Directly mailed HPVSS vs HPVSS supported by CHW Device: Not reported	Uptake at 6 months	81.0% (242/300) SS supported by CHW and 71.6% (214/300) among directly mailed SS group (*p* < 0.001).	Directly mailed kits included a return envelope. Mailed HPVSS group also had brief health education conversation. 30 min in-person study visits by a CHW at a community location. CHW provided health education and instructions on HPVSS.
MacDonald et al., 2021,^58^ New Zealand	Design: RCT (cluster) Aim: To increase cervical screening for under-screened/never-screened Māori women	*n* = 931 Māori Women aged 25–69 Non-attenders (last screened more than 4 years ago)	HPVSS offered in-clinic (could be done at clinic, home or community centre) vs cervical smear (usual care) Device: Nylon-flocked swab (Copan Floxswab)	Uptake	HPVSS in-clinic 254/500 (50.8%) vs control cervical smear 94/431 (21.8%) (*p* < 0.001). 217/500, 43.4% who were offered HPVSS had only HPVSS.	Women offered HPVSS by healthcare worker in the clinics. Women in the intervention group were significantly younger and more likely to be living in deprived areas. Women not consented for smear (usual practice) but consented for HPVSS. Rural setting. Follow-up not clearly recorded.
Molokwu et al., 2015,^51^ USA	Design: RCT Aim: To evaluate the effect of a community outreach worker (promotora)-led high intensity educational intervention compared with control without promotora-led intervention on CC screening preference.	*n* = 201 Hispanic women aged 30–65 Non-attenders (no cervical screening in the last 3 years)	Culturally tailored education by CHW vs written education pamphlet Device: Not reported	Acceptability Preference	The HPVSS acceptability score was 25.02 in culturally tailored education CHW group vs 24.06 written pamphlet education group (*p* = 0.039). 28.6% who had culturally tailored education vs 35.1% who had written pamphlet education preferred self- sampling (*p* = 0.536).	All women did HPVSS. Not all the women did Pap smear.
Ngu et al., 2022,^65^ Hong Kong	Design: Prospective cohort Aim: The aim of this study was to assess the effectiveness of HPVSS for CC screening and the best means of service delivery, with a specific focus on under-screened women, particularly during the COVID-19 pandemic	*n* = 187 Chinese immigrants and Filipino domestic helpers Women aged between 30 and 66 Non-attenders (no screening in the last 3 years)	Mailed HPVSS kit, then invited for clinician-based sampling Device: Dacron swab	Uptake Acceptability Ease Confidence	162/187 (86.6%) returned a HPVSS kit vs 98/187 (52.9%) for clinician-based sampling. Face-to-face methods had a 65.5% recruitment rate (173/264), whereas online methods had 7.9% (14/177). 67.1% found HPVSS easy/very easy and 71.3% were confident/very confident with HPVSS in Filipino domestic helpers. 52.9% found HPVSS easy/very easy and 49.3% were confident/very confident with HPVSS in Chinese immigrants. No statistical testing reported.	Women received an educational health talk. If participants attended face-to-face talk, they were given HPVSS kit. If they attended online talks, the kit was posted. After returning kit, they were then invited for clinician-based sampling. Used non-governmental organisation to recruit women. Follow-up period not clearly recorded.
Sewali et al., 2015,^52^ USA	Design: RCT Aim: To examine the difference in successful test completion rates between home-based HPV tests and clinic-based Pap tests. To see if this innovative testing method might improve CC screening rates in this particular underserved population	*n* = 63 Somali women aged 25–70 Non-attenders (no Pap smear in the last 3 years)	Home-based HPVSS supported by CHWs vs standard Pap test Device: Not reported	Uptake at 3 months	Home-based HPVSS with CHW (21/32, 65.6%) vs Standard Pap Clinic (6/31, 19.4%) *p* = 0.0002.	Used Somali CHWs to provide educational session about HPVSS and the kit before screening. Somali immigrants living in the USA for 10 years or less.
Waller et al., 2006,^53^ UK	Study design: Cross-sectional questionnaire Aim: To find out whether self-sampling would be acceptable to women if carried out on their own, with only a written instruction sheet and no additional information from clinicians.	*n* = 100 Black, Asian, ‘other’ ethnicity women aged 19–65	In-clinic HPVSS vs clinician-based sampling Device: Digene kit	Acceptability Attitudes	No differences in attitudes to the clinician-administered test by ethnicity, but there were for self-sampling. Asian women had the most negative attitudes (mean score = 9.10, 95% CI 8.10 to 10.11), while White women had the most positive attitudes (mean score 7.90, 95% CI 7.76 to 8.05). Differences between ethnic groups were significant (*F*_(4,849) _= 2.71, *p* = 0.03).	Women took their own sample then had a clinician-administered HPV test and cervical smear. Women received written instructions on how to complete HPVSS and no other guidance. Women were already attending for a smear test. Higher score correlates with more negative attitudes. Scores were a sum of scales for embarrassment, discomfort, unpleasantness, anxiety, relaxation (reverse scored) and confidence (reverse scored).
**Older women**						
Gök et al., 2012,^42^ The Netherlands	Design: Pooled data from two randomised controlled trials (RCTs)^43,44^ Aim: Aim not explicit. The authors analysed which subpopulations among non-attendees are targeted by HPV self-sampling, and which characteristics relate to hrHPV prevalence and yield of CIN2/CIN3.	*n* = 11,053 (over 54) (*n* = 52,447 overall) Data have been extracted for women aged 54–63 Non-attenders (did not attend screening after 2 invitations)	Directly mailed HPVSS vs usual screening reminders for clinician-based sampling Devices: Lavage-based Delphi, Viba brush	Uptake at 12 months	54–58 years: 28% (1637/5806) HPVSS vs 69% (*n* = 20,181) regular screening programme. 59–63 years: 27% (1485/5429) HPVSS vs 59% (*n* = 22,694) in regular screening.	Pooled data from two RCTs – same design apart from screening tool: one study used lavage-based Delphi, one RCT used Viba brush. Comparison is between non-attenders and those who participated in regular screening programme. Women were sent a letter informing them they would receive either a HPVSS kit or invitation for cytology.
Kellen et al., 2018,^62^ Belgium	Design: RCT Aim: To evaluate the effectiveness of two different strategies of offering HPVSS to women who do not participate in the Flemish screening programme, compared with standard recall letter or no intervention at all	*n* = 19,816 (over 50) *n* = 35,354 overall Data have been extracted for women aged 50–64 Non-attenders (no screening since 2008)	Directly mailed HPVSS vs opt-in (letter sent with offer to order HPVSS kit by phone, mail or website) vs regular recall letter for clinician-based screening vs no intervention Device: Qvintip brush	Uptake at 12 months	All Over 50s: Mail to all: 20.06% (1017/5069) vs opt in: 11.84% (603/5093) vs recall letter: 6.67% (323/4841) vs no intervention: (4.8% 231/4813). Uptake by age categories: 50–54: (1) HPVSS mailed: 19.3%, 235/1219 (2) Opt-in letter: 12.3%, 154/1253 (3) Recall letter: 8.5%, 1055/1233 (4) No intervention: 7.1%, 88/1237 55–59 (1) HPVSS mailed: 20.4%, 308/1511 (2) Opt-in letter: 12.0%, 181/1510 (3) Recall letter: 6.8%, 99/1452 (4) No intervention: 4.6%, 67/1472 60–64 (1) HPVSS mailed: 20.3%, 474/2339 (2) Opt-in letter: 11.5%, 268/2330 (3) Recall letter: 5.5%, 119/2156 (4) No intervention: 3.6%, 76/2104	Main findings extracted. Study conducted as part of the Flemish screening programme. Directly mailed HPVSS kit had pre-paid envelope. Those who were sent a kit or opt-in letter were sent a reminder after 4 years.
Landy et al., 2022,^54^ UK	Design: RCT Aim: To assess if offering non-speculum clinician taken sampling and self-sampling increases uptake for lapsed attenders aged 50–64.	*n* = 784 Women aged 50–64 Non-attenders (last screened 6–15 years before study)	Opt-in letter (order HPVSS postal kit or op for non-speculum clinician sampling) vs control (written reminder for clinician-based sampling) Device: Flocked swab FLOQSwab	Uptake at 4 months Acceptability Discomfort Perceived efficacy Embarrassment Preference	80/393 (20.4%) opt-in group vs 19/391 (4.9%) control were screened (*p* < 0.001). 37/80 (46.3%) opted for HPVSS, 28.8% (23/80) for non-speculum, 25% (20/80) for speculum in intervention group. 88.1% HPVSS vs 83.3% Pap excellent/good experience with test, *p* = 0.735. HPVSS more discomfort than clinician non-speculum (42.9% vs 38.9%) *p* = 0.775. 95.1% HPVSS were fairly/very confident in test done properly compared to 94.1% in non-speculum (*p* = 1). 35.7% HPVSS were fairly/very confident in test accuracy compared to 76.5% in non-speculum (*p* = 0.009). 4.8% HPVSS reported feeling embarrassed, vs 27.8% non-speculum (*p* = 0.021). 70.6% of non-speculum group said they would opt for non-speculum, 90.5% of HPVSS group said they would opt for HPVSS (*p* < 0.001). Sub-analysis by ethnicity: Selection of screening test differed by ethnicity in the intervention arm (*p* < 0.001). Half the screened women from White backgrounds self-sampled (50.7%, *n* = 38/75), whereas the majority of women from Asian (53.3%, *n* = 8/15), Black (71.4%, *n* = 15/21) and mixed/other/unknown backgrounds (66.7%, *n* = 6/9) attended speculum screening. (72.2% (13/18) non-speculum, 88.1% (37/42) self-sampling) ‘agreed’ or ‘strongly agreed’ that it was important to have a choice of tests.	Usual care was written reminder. Women who ordered self-sampling kits had a kit posted to their home address. Questionnaire was attached to the HPVSS kit.
Sultana et al., 2016,^61^ Australia	Design: RCT Aim: To determine whether HPVSS could increase participation in the Australian cervical screening programme	Reported by group: Aged 50–59: 3311 Aged 60–69: 3899 Lowest SES: 3266 (*n* = 16,320) overall Women aged 30–69 under screened and never screened	Mailed HPVSS vs standard reminder letter. Device: Flocked swab (Copan Italia, Brescia, Italy)	Uptake at 6 months	Age 50–59 and under screened: 13.6% HPVSS vs 7.3% Pap. difference 6.30 (95% CI 2.40 to 10.30). Age 50–59 and never screened: 18.4% HPVSS vs 6.1% Pap. difference 12.30 (95% CI 8.40 to 16.20). Age 60–69 and under screened: 17.9% HPVSS vs 8.3% Pap. difference 9.60 (95% CI 4.40 to 14.90). Age 60–69 and never screened: 14.2% HPVSS vs 3.5% Pap. difference 10.70 (95% CI 8.30 to 13.00). Sub-analysis for SES: Lowest SES quintile and never screened: 20.2% HPVSS vs 6.1% Pap. difference 14.10 (95% CI 10.40 to 17.80). Lowest SES quintile and under screened: 12.1% HPVSS vs 5.6% Pap. Difference 6.40 (95% CI 2.80 to 10.10).	Under screened defined as not screened in the past 5 years. Never screened defined as women on the electoral roll who were not matched on the registry. Also reported for deprivation quintiles.
Virtanen et al., 2011,^59^ Finland	Design: RCT Aim: To assess the effects of a hrHPV self-sampling test on increasing the attendance and coverage of CC screening in comparison to a written reminder letter	*n* = 1456 women over 50 *n* = 12,839 overall (women aged 30–65)	Directly Mailed HPVSS kits vs standard reminder letter Device: Delphi screener	Uptake	Mailed HPVSS 33.75% (135/400) vs 28.7% (303/1056) for reminder letter. Age categories: 50–54: (1) HPVSS: 42/146, 28.8% (2) Reminder letter 131/393, 33.3% 55–59 (1) HPVSS: 52/139, 37.4% (2) Reminder: 86/341, 25.6% 60–64 (1) HPVSS: 41/115, 35.7% (2) Reminder letter 86/322, 26.7%	Both groups had a second reminder. Study conducted as part of screening programme.
Virtanen et al., 2015,^60^ Finland	Design: Prospective cohort Aim: To study the effect of reminder letters (1st reminder) and self-sampling tests (2nd reminder) on attendance	*n* = 16,111 women over 50 Non-attenders (after two invitations for cervical screening) *n* = 31,053 overall (women aged 30–60)	Mailed HPVSS vs standard screening after two invitations Device: Delphi screener	Uptake	All over 50s: 19.7% (331/1676) HPVSS vs 85.0% (12,264/14,435) standard screening Age categories: 50–54: 18.1% (97/535) self-sampling vs 84.4% (3782/4483) regular screening 55–59: 23.3% (131/562) self-sampling vs 84.5% (4001/4735) regular screening 60–64: 19.5% (113/579) vs 85.9% (4481/5217) regular screening	Study conducted as part of routine screening programmes.
Wedisinghe et al., 2022,^55^ UK	Design: Prospective cohort Aim: To assess the impact of offering multiple screening options to the target population (women whose screening was overdue) and to determine the effect of different factors on screening uptake	*n* = 648 (aged 56–60) *n* = 4146 overall Women aged 56–60, non-attenders (had not been screened in the last 3.5 years)	Mailed HPVSS kit vs opt-in (letter with option of HPVSS, or clinician sampling) vs routine screening Device: Evalyn brush	Uptake at 4 months	Mailed kits 9% vs opt-in HPVSS 8%. Further 6% opted for GP/Hospital screening after receiving opt-in letter. Overall screening participation: Mailed HPVSS kit 12% (95% CI 9 to 17), opt-in 15% (95% CI 11 to 20), standard recall 3% (95% CI 0 to 11).	
**Younger women**						
Kitchener et al., 2018,^56^ UK	Design: RCT (cluster) Aim: To measure the feasibility and effectiveness of interventions to increase cervical screening uptake among young women	*n* = 10,126 Women invited for their first cervical screen (aged 24 years and 6 months) Non-attenders (not booked/attended first cervical screening appointment)	Mailed HPVSS kit vs opt-in vs nurse navigator for Pap smear vs timed appointment for Pap smear vs choice between nurse navigator or self-sampling kit vs control (invitation letter) Device: Delphi lavage or Evalyn brush	Uptake at 12 months	Control (invitation letter for Pap smear): 16.2% (613/3782). Mailed HPVSS kit sent: 243/1141 (21.3%) had been screened (*p* = 0.001 against control). Opt-in (HPVSS kit available on request): 209/1290 (16.2%) (*p* = 0.505 against control). Nurse navigator (for Pap smear): 146/1007 (14.49%) (*p* = 0.401 against control). Timed appointment: 323/1629 (19.8%) (*p* = 0.001 against control). Choice (between nurse navigator or self-sample) group: 240/1277 (18.8%) (*p* = 0.466 against control).	Phase 1 evaluated the use of a patient information leaflet and opportunity to book cervical screen appointment online. These results report on non-responders to phase 1 of the trial. *p* value represents comparison of intervention with control (standard invitation letter)

CIN: cervical intraepithelial neoplasia.

### Quality assessment

The overall quality of the RCTs was high (Table 3). The majority of the concerns of bias in these studies were due to poor reporting of allocation concealment. Due to the nature of the intervention, participants were unable to be blinded. According to ROBINS-I, four studies were ‘moderate’, four studies were ‘serious’ and one study was ‘low’ risk of bias (Table 4). The results have been synthesised for each outcome and each underserved group.

**Table 3.** Risk of bias in randomised controlled trials.

Source of bias	Randomisation	Deviations from interventions	Missing outcome data	Measurement	Reporting	Overall
Author, year
Brewer et al., 2021^57^	Low	Low	Low	Low	Low	Low
Carrasquillo et al., 2018^48^	Low	Low	Low	Low	Low	Low
Gök et al., 2011^a^ ^43^	Some concerns	Low	Low	Low	Some concerns	Some concerns
Gök et al., 2010^a^ ^44^	Some concerns	Low	Low	Low	Low	Some concerns
Kellen et al., 2018^62^	Some concerns	Low	Low	Low	Low	Some concerns
Kitchener et al., 2018^56^	Low	Low	Low	Low	Low	Low
Kobetz et al., 2018^50^	Some concerns	Low	Low	Low	Low	Some concerns
Landy et al., 2022^54^	Low	Low	Low	Low	Some concerns	Some concerns
Lazcano et al., 2011^68^	Some concerns	Low	Low	Low	High	High
MacDonald et al., 2021^58^	Some concerns	Low	Low	Low	Low	Some concerns
Molokwu et al., 2018^51^	Some concerns	Low	Low	High	Low	High
Pretsch et al., 2023^47^	Low	Low	Low	Low	Low	Low
Sancho et al., 2013^64^	Some concerns	Low	Low	Low	Some concerns	Some concerns
Sewali et al., 2015^52^	Low	Low	Low	Low	Low	Low
Sultana et al., 2016^61^	Low	Low	Low	Low	Some concerns	Some concerns
Tranberg et al., 2018^63^	Low	Low	Low	Low	Low	Low
Virtanen et al., 2011^59^	Some concerns	Low	Low	Low	Low	Some concerns

^a^ Reported as Gök et al., 2012.^42^

**Table 4.** Risk of bias in non-randomised studies.

Source of bias	Confounding bias	Selection of participants	Intervention classification	Deviations from intended interventions	Missing data	Outcome measurement	Selection of results	Overall
Author, year
Anand et al., 2022^66^	Moderate	Low	Low	Low	Low	NI	NI	Moderate
Anhang et al., 2005^45^	Low	Low	Low	Low	Low	Serious	Serious	Serious
Castle et al., 2011^49^	Serious risk	Serious risk	Low	Low	Low	NI	Low	Serious
Kilfoyle et al., 2018^46^	Low	Moderate	Low	Low	Low	Serious	Low	Serious
McFarlane et al., 2022^67^	Moderate	Low	Low	Low	NI	Serious	Moderate	Serious
Ngu et al., 2022^65^	Moderate	Low	Low	Low	Low	Low	Low	Moderate
Virtanen et al., 2015^60^	Low	Low	Low	Low	Low	NI	Low	Low
Waller et al., 2006^53^	Moderate	Low	Low	Low	Low	Serious	Low	Moderate
Wedisinghe et al., 2022^55^	Moderate	Low	Low	Low	Low	Low	Low	Moderate

NI: no information.

### Low socioeconomic groups

Ten studies reported on women from low-SES groups.^45–47,57,61,63,64,66–68^ Eight studies reported low-SES groups as their main population^45–47,63,64,66–68^ and two studies performed a sub-analysis for SES.^57,61^ The criteria for low SES varied across studies, but generally participants were identified based on income, accessing free healthcare or living in a deprived area.

#### Uptake

Four RCTs found mailing HPVSS kits to non-attenders increased uptake compared to a reminder for clinician-based screening: 31.4% vs 19.5%,^63^ 18.3% vs 2%, *p* < 0.001,^64^ 8.7% vs 3%, *p* = 0.0015^57^ and 20.2% vs 6.1% for never-screened, 12.1% vs 5.6% for under-screened.^61^ One of these RCTs found directly mailed kits resulted in higher uptake compared to an opt-in approach where women from low-SES groups were invited to order their own kits via website, email or phone: 31.4% vs 24.7%, participation difference = 6.7, 95% confidence interval (CI) 2.8 to 10.6.^63^

One RCT found mailing HPVSS to low-SES women and offering assistance with scheduling in-clinic appointments increased uptake compared to scheduling assistance alone (72% vs 37%, relative risk 1.93, 95% CI 1.62 to 2.31).^47^

Community nurses conducting home visits to offer HPVSS in an RCT resulted in significantly higher uptake than written invitations for clinician-based screening (98% vs 87%, *p* = 0.001).^68^ However, the study was deemed to have a high risk of bias (Table 3); the analysis was ‘per-protocol’ and excluded women who were not at home during recruitment for HPVSS.

Anand et al. found uptake to be higher in a group that received an in-person health education session than a group that received written pamphlet education (98% vs 82%).^66^

#### Acceptability

Two studies compared the acceptability of HPVSS with clinician-based sampling, both of which were deemed to have serious risk of bias (Table 4).^45,46^ Kilfoyle et al. mailed HPVSS kits directly to low-income women.^46^ There was no significant difference between HPVSS and clinician-based sampling groups on attitudes towards test (81% vs 75%, *p* = 0.353), willingness to repeat (98% vs 98%, *p* = 1.00) or perceived safety of the test (99% vs 97%, *p* = 0.317).^46^ However, pain and discomfort were higher for clinician-based sampling (43% vs 18%, *p* < 0.001 and 30% vs 8%, *p* = 0.001, respectively). HPVSS was more convenient (13% vs 31%, *p* = 0.003) and more women expressed a preference for HPVSS than clinician-based sampling (51% vs 19%, *p* < 0.0001).^46^ Anhang et al. offered HPVSS in-clinic along with clinician-based sampling.^45^ Having had both tests, of the women who indicated a preference, more preferred clinician-based sampling than HPVSS (68% vs 32%) but no statistical test was reported.^45^

Two observational studies reported on the acceptability of HPVSS when given different supporting materials for HPVSS for low-SES women.^66,67^ Anand et al. found that an in-person health education session compared to a leaflet resulted in HPVSS being reported as more acceptable (100% vs 94%) and produced a greater uptake rate (98% vs 92%) of HPVSS, although there was no *p*-value stated.^66^ McFarlane et al. found there was no difference in the acceptability or perceived efficacy of HPVSS between using a culturally tailored kit (which included illustrations and colours designed to appeal to Jamaican women), compared to a ‘plain’ kit (*p* = 0.09).^67^ However, the ‘plain’ kit received more positive attitudes than the kit which was culturally targeted (*M* = 6.24 vs 5.86) (Table 2).^67^

### Women from minority ethnic backgrounds

Nine studies were conducted among minority ethnic groups.

#### Uptake

Seven studies looked at uptake in ethnic minority groups. Two RCTs found that offering HPVSS in a clinic to Māori women^58^ or Māori, Pacific and Asian women^57^ in New Zealand resulted in greater uptake compared to offering clinician-based sampling: 6.4% vs 2.7%, *p* = 0.002^57^ and 50.8% vs 21.8%, *p* < 0.001.^58^ MacDonald et al. offered women HPVSS in a clinic; however, women were also allowed to complete the test at home.^58^ One of these studies found higher uptake for the group mailed HPVSS kits compared to those given a written invitation to clinician-based sampling (14% vs 2.7%, *p* < 0.0001).^57^

Three RCTs evaluated the use of HPVSS supported by a community health worker (CHW) among Black, Haitian and Hispanic women^48,50^ and Somali women^52^ in the USA. The uptake of HPVSS supported by a CHW was significantly higher than mailing HPVSS kits (81.0% vs 71.6%, *p* < 0.001)^50^ or offering clinician-based sampling (65.6% vs 19.4%, *p* = 0.002).^52^ HPVSS supported by CHWs also resulted in higher uptake compared with using nurse navigators for clinician-based sampling (77.3% vs 42.5%, *p* < 0.001) or an educational outreach session (77.3% vs 31.3%, *p* < 0.01).^48^

One study set in the USA found that after being given the choice between HPVSS or clinician-based sampling, African-American women were more likely to complete screening using home-based HPVSS than clinician-based sampling (80.5% vs 40.5%, *p* = 0.0001).^49^ This study was deemed to have serious risk of bias (Table 4).

Ngu et al. found mailing HPVSS kits to Chinese immigrants and Filipino domestic helpers in Hong Kong after an education session had higher uptake compared to offering clinician-based sampling (86.6% vs 52.9%). Recruitment of women was greater using face-to-face recruitment compared to online methods (65.5% vs 7.9%); no *p*-value was reported.^65^

#### Acceptability

A culturally tailored educational intervention for Hispanic women in the USA resulted in higher acceptability of HPVSS compared to an educational leaflet (acceptability score 25.02 vs 24.06, *p* = 0.039) but there was no effect on preference between self-sampling and clinician-collected sampling (28.6% vs 35.1%, *p* = 0.536).^51^ This study was deemed to have high risk of bias due to the varying measurement of acceptability (Table 3).

Four studies reported differences between ethnic groups as sub-analysis.^45,46,53,54^ Women who were not Hispanic were more likely to prefer HPVSS than clinician sampling, than those women who were Hispanic (48.6% vs 28.5%, *p* < 0.05).^45^ Kilfoyle et al. reported that Black women and women other than White were less likely than White women to prefer HPVSS over clinician-based sampling (odds ratio 0.52, 95% CI 0.30 to 0.92).^46^ Landy et al. reported that half of the screened White women selected HPVSS (50.7%); however women from Black (71.4%), Asian (53.3%) and mixed/other/unknown backgrounds (66.7%) were more likely to choose clinician-based sampling (*p* < 0.001).^54^ Furthermore, a UK study reported that Asian women had negative attitudes towards self-sampling but White women had more positive attitudes (mean score 9.10 vs 7.90, *p* = 0.03).^53^

### Younger women

Only one relevant study was identified which included younger women (aged 25) and it examined uptake^56^ of initial screening invitations; no studies reported on acceptability of HPVSS for younger women. Therefore the results presented here cannot be generalised to young women.

#### Uptake

Kitchener et al. conducted an RCT in the UK and reported greater uptake for mailing HPVSS kits (21.3%) and timed appointments for clinician-based sampling (19.8%) compared with standard written reminder letters (16.2%; *p* = 0.001 and *p* = 0.01, respectively).^56^ The opt-in approach for HPVSS resulted in an equivalent uptake rate to standard written reminders (16.2% vs 16.2%, *p* = 0.505).^56^

### Older women

Seven studies were conducted among older women.

#### Uptake

Seven studies reported on the uptake of HPVSS among older women (≥50 years).^42,54,55,59–62^ One of these exclusively included older women.^54^ The other six studies stratified results by age; therefore data could be extracted from results tables in the studies, but significance testing using *p*-values was not reported for these.^42,55,59–62^

Three studies found that directly mailed kits had higher uptake than standard recall for clinician-based sampling^59,61,62^ (Table 2). Two studies found directly mailed kits had higher uptake than using an opt-in approach.^55,62^ The opt-in approach in one of these studies included both clinician sampling and HPVSS.^55^ Two further studies found mailed HPVSS kits generated additional uptake in non-attenders who did not engage with clinician-based screening after two invitations (Table 2).^42,60^ The statistical significance of these results were not reported.

An opt-in approach resulted in higher uptake than standard recall: 20.4% vs 4.9%, *p* < 0.001^54^ and 15% vs 3%.^55^ The opt-in approach in both studies included the option of HPVSS or standard clinician sampling; thus the uptake for opt-in was not exclusively for HPVSS.

#### Acceptability

There was no significant difference in the acceptability, perceived efficacy or discomfort associated with HPVSS compared to clinician-based sampling (Table 2).^54^ However, HPVSS was less embarrassing (4.8% vs 27.8%, *p* = 0.021) and was preferred to clinician-based sampling (90.5% vs 70.6%, *p* < 0.001).^54^ Women had less confidence in HPVSS accuracy (35.7% vs 76.5%, *p* = 0.009). Women from both groups (offered self-sampling and clinician-based sampling) agreed it was important to have a choice about sampling options (Table 2).^54^

## Discussion

Overall, opt-out methods, such as directly mailing HPVSS kits and using CHWs to offer HPVSS, resulted in higher uptake of cervical screening compared to opt-in strategies for HPVSS or offering clinician-based sampling among most women. This review has shown higher uptake when offering underserved groups the option of HPVSS compared to clinician-based sampling. Although not as effective as the opt-out strategies, offering women the choice to opt-in for HPVSS still resulted in higher uptake than clinician-based sampling, particularly for some underserved groups. Although overall HPVSS was acceptable for underserved groups, differences were observed in the acceptability and preferences of screening modality between different ethnic groups. There was some variation in HPVSS acceptability based on how it was implemented (opt in/opt out), but the evidence was limited.

### What is optimal: opt-in or opt-out

In relation to opt-out strategies, directly mailing HPVSS kits resulted in significantly higher uptake of HPVSS in low-SES groups, ethnic minority groups and younger women compared with an invitation or reminder for clinician-based sampling.^56,57,64^ This finding aligns with a recent meta-analysis showing a two-fold increase in screening uptake when HPVSS kits were mailed directly to women, as opposed to inviting them to clinician-based screening.^29^ Mailing kits has been found to increase screening uptake in both non-attenders and the general population.^29,69^ This review builds on this evidence by confirming that directly mailing kits improves uptake in a range of underserved groups. Directly mailing kits can mitigate barriers such as accessing kits, embarrassment and inconvenience of in-clinic sampling.^31^ The levels of uptake seen with directly mailed kits were higher than the uptake achieved by an opt-in approach and offer of clinician sampling.^56,57^

Although not as effective as the opt-out strategies, offering women the choice to opt-in for HPVSS still resulted in higher uptake than clinician-based sampling particularly for individuals who were older^54^ and those from low-SES backgrounds.^63^ In older women there was significantly greater uptake for an opt-in approach for HPVSS or non-speculum clinician sampling compared to clinician-based sampling.^54^ This contrasts with results from Arbyn et al. who found opt-in approaches to not significantly increase uptake compared to routine invitations for clinician-based sampling.^12^ However, there was a lack of literature focusing on young women. Only one study was identified and it focused on attendance to first screening invite in the UK. It found no difference between an opt-in approach for HPVSS and clinician-based sampling.^56^ It is important that further research focusing on young women is to be conducted to explore preference for opt-in/opt-out choice of HPVSS.

### Further strategies

As well as offering HPVSS, further strategies to increase uptake were explored in some studies, in particular those focusing on women from lower SES and ethnic minority backgrounds. A variety of alternative strategies were used, such as CHWs, an in-clinic HPVSS option and educational materials. All studies that reported on CHWs found significantly higher uptake compared to alternative strategies among ethnic minority and low-SES groups.^48,50,52,68^ Kobetz et al. found significantly higher uptake for HPVSS supported by a CHW than directly mailed kits.^50^ Two studies found significantly higher uptake for an in-clinic HPVSS option compared to clinician-based sampling in ethnic minority women.^57,58^ One of these studies offered in-clinic HPVSS for women to do either in clinic or at home.^58^

### HPVSS modality acceptability

HPVSS was acceptable for underserved groups. However, differences were observed in the acceptability and preferences of screening modality between different ethnic groups. Hispanic women and Black women were less likely to prefer self-sampling to clinician-based sampling compared to White women.^45,46^ Black, Asian and women of other ethnic backgrounds were more likely to opt for clinician-collected samples than White women.^54^ The cultural appropriateness and perceived lack of reliability of self-collected samples have been identified as barriers for uptake among ethnic minority women.^70^ Three of the four studies reporting on CHWs in ethnic minority groups invited women to an education session pre-testing.^48,50,52^

Cultural tailoring improved acceptability of HPVSS in a group of Hispanic women^51^ whereas no difference was found in a group of Jamaican women^67^ from low-income backgrounds. The study which showed a significant difference was at high risk of bias, thus questioning confidence in the finding.^51^ In another study higher levels of HPVSS acceptability were suggested for an in-person health education session compared with written education for women from a low-income setting (statistical significance not reported).^66^

### Recommendations for introduction of HPVSS modality

In addition to the modality of offering HPVSS, this review suggests that access to support from healthcare professionals (HCPs) and education about screening are important components for ensuring that implementation of HPVSS is planned in a manner that will aid equitable access to cervical screening. A previous review found the home to be a highly acceptable setting for HPVSS, but women were found to prefer clinic-based sampling if sampling in the home meant they would not have access to an HCP.^26^ Using CHWs combines the benefits of home-based self-sampling with the support and reassurance offered by a trusted professional. CHWs are uniquely placed to reassure the women of their communities. Face-to-face interaction and education provided by CHWs is likely to alleviate concerns women have about self-sampling, such as lack of confidence in completing the kit.^70^ Careful identification of suitable communities is necessary for implementing this approach in large-scale CSPs. High cost implications and practical considerations need to be evaluated within the NHS setting. CSPs might be most suitable for targeted interventions, such as CHWs, where particularly low levels of screening uptake within certain communities are identified.

### Strengths of this research

We applied a critical lens to understanding what works best and for which individuals when planning the introduction of HPVSS into CSPs. The UK has recently trialled the use of HPVSS for increasing cervical screening among non-attenders as well as an option to complete a screening in clinic.^35^ The findings from this study can directly inform plans for introduction of HPVSS in the UK and in other nations with an organised screening programme.

Most studies within this review were RCTs (with low or some concerns about bias). All studies reporting significance between implementation strategies were RCTs, most of which were high quality and well reported (Table 3). Studies reporting the acceptability of HPVSS based on hypothetical scenarios were excluded; therefore the findings reflect the views of participants who were actually offered screening (HPVSS or clinician sampling). Given the multifaceted and complex nature of implementation strategies within the included studies, a narrative synthesis was used to provide a comprehensive knowledge summary to understand how HPVSS implementation strategies work in different contexts, for different groups. This review is unique as it has looked at which underserved groups might benefit most from HPVSS and the best methods for implementing HPVSS to achieve maximum benefit.

### Limitations

It is important to note that not all underserved groups were represented in this review and therefore findings cannot be assumed as applicable; no studies were identified that reported on LGBTQ+ groups, or women with physical disabilities or learning disabilities. Furthermore, only one study reported on younger women, and thus our results cannot be applied to young women broadly. Results focusing on older women were mostly extracted by sub-group data from studies looking at the general population, and the statistical significance of these findings was not reported in the individual studies. This limits the strength of our conclusions for the group of older women. Several other studies did not report significance testing. In their raw form, such results provide some insight into the effectiveness of implementation strategies; however the strength of conclusions that can be drawn from these studies is greatly limited.

There was limited evidence for the secondary objective of this review (to understand the impact of implementation strategies on acceptability of HPVSS) with only three studies examining the impact of different strategies for implementing HPVSS on acceptability.^51,66,67^

Many of the other studies compared HPVSS acceptability with clinician-based sampling acceptability. Further research is needed to explore whether and how implementation strategies can influence HPVSS acceptability.

### Recommendations

The optimum implementation strategies for each group are summarised in Table 5.

**Table 5.** Summary of key findings for each group.

Underserved group	Optimum HPVSS implementation method(s)
Low socioeconomic status groups	Directly mailed kits Community health workers In-person health education session
Ethnic minority groups	Community health workers Directly mailed kits Culturally tailored health education sessions
Older women	Directly mailed kits

Directly mailing HPVSS kits as part of the UK CSP should be considered for reaching underserved groups. Examination of appropriate supporting materials and health education promotion components to include with directly mailed kits is important to optimise uptake.

As seen by the success of CHWs, the face-to-face engagement of women from underserved groups remains important. Ensuring women have access to HCPs and are well supported is important for the implementation of HPVSS.

Although inferior to directly mailing kits, offering HPVSS in clinic and opt-in approaches should not be dismissed. Several studies observed an increase in uptake using opt-in approaches compared to standard screening pathways. This increase was seen in younger women (although only one study), women from ethnic minority backgrounds, older women and low-SES group (one study). Opt-in and in-clinic strategies are more economical when considering implementation on a national level.^31^

It is essential to consider the needs of underserved groups to minimise screening inequalities and to promote uptake.^71^ Given the paucity of evidence for some underserved identified by this review, future work should examine the implementation of HPVSS among those with LGBTQ+ identities, learning disabilities, physical disabilities and among younger women.

## Conclusion

To facilitate an equitable and person-centred approach to HPVSS implementation, the offer of opt-out HPVSS methods (e.g. directly mailing HPVSS kits) should be considered a priority as it has the potential to increase uptake of cervical screening in underserved groups. Screening uptake may further improve in women from low-SES and ethnic minority background groups when CHWs are used within HPVSS implementation.

## Supplemental Material

sj-docx-1-msc-10.1177_09691413241274312 - Supplemental material for Identifying optimum implementation for human papillomavirus self-sampling in underserved communities: A systematic review Supplemental material, sj-docx-1-msc-10.1177_09691413241274312 for Identifying optimum implementation for human papillomavirus self-sampling in underserved communities: A systematic review by Olivia Mackay, Kate Joanna Lifford, Anahat Kalra and Denitza Williams in Journal of Medical Screening

sj-docx-2-msc-10.1177_09691413241274312 - Supplemental material for Identifying optimum implementation for human papillomavirus self-sampling in underserved communities: A systematic review Supplemental material, sj-docx-2-msc-10.1177_09691413241274312 for Identifying optimum implementation for human papillomavirus self-sampling in underserved communities: A systematic review by Olivia Mackay, Kate Joanna Lifford, Anahat Kalra and Denitza Williams in Journal of Medical Screening

sj-docx-3-msc-10.1177_09691413241274312 - Supplemental material for Identifying optimum implementation for human papillomavirus self-sampling in underserved communities: A systematic review Supplemental material, sj-docx-3-msc-10.1177_09691413241274312 for Identifying optimum implementation for human papillomavirus self-sampling in underserved communities: A systematic review by Olivia Mackay, Kate Joanna Lifford, Anahat Kalra and Denitza Williams in Journal of Medical Screening

## References

[1] Cancer Research UK. Cancer incidence by age, https://www.cancerresearchuk.org/health-professional/cancer-statistics/incidence/age#heading-Two (2021, accessed 4.3.2023).

[2] Castellsague X . Natural history and epidemiology of HPV infection and cervical cancer. Gynecol Oncol 2008; 110: S4–S7. Research Support, Non-U.S. Gov’t Review. 10.1016/j.ygyno.2008.07.045 18760711

[3] Zhang S Xu H Zhang L , et al. Cervical cancer: epidemiology, risk factors and screening. Chin J Cancer Res 2020; 32: 720–728. 33446995 10.21147/j.issn.1000-9604.2020.06.05 PMC7797226

[4] Nour NM . Cervical cancer: a preventable death. Rev Obstet Gynecol 2009; 2: 240–244. 20111660 PMC2812875

[5] UK Health Security Agency. HPV vaccination guidance for healthcare practitioners (version 6), https://www.gov.uk/government/collections/hpv-vaccination-programme (2022, accessed 4.3.2023).

[6] Falcaro M Castañon A Ndlela B , et al. The effects of the national HPV vaccination programme in England, UK, on cervical cancer and grade 3 cervical intraepithelial neoplasia incidence: a register-based observational study. Lancet 2021; 398: 2084–2092. 34741816 10.1016/S0140-6736(21)02178-4

[7] Public Health England. Cervical screening: primary HPV screening implementation, https://www.gov.uk/government/publications/cervical-screening-primary-hpv-screening-implementation (2019, accessed 13.11.2022).

[8] NI Direct. Cervical screening, https://www.nidirect.gov.uk/conditions/cervical-cancer (accessed 24.10.2022).

[9] Public Health Wales. Cervical screening Wales, https://phw.nhs.wales/services-and-teams/cervical-screening-wales/what-is-cervical-screening/ (accessed 24.10.2022).

[10] NHS Inform Scotland. Cervical screening (smear test), https://www.nhsinform.scot/healthy-living/screening/cervical/cervical-screening-smear-test (2022, accessed 24.10.2022).

[11] Landy R Pesola F Castanon A , et al. Impact of cervical screening on cervical cancer mortality: estimation using stage-specific results from a nested case-control study. Br J Cancer 2016; 115: 1140–1146. 27632376 10.1038/bjc.2016.290 PMC5117785

[12] Arbyn M Smith SB Temin S , et al. Detecting cervical precancer and reaching underscreened women by using HPV testing on self samples: updated meta-analyses. Br Med J 2018; 363: k4823. 20181205. 10.1136/bmj.k4823 PMC6278587 30518635

[13] Costa S Verberckmoes B Castle PE , et al. Offering HPV self-sampling kits: an updated meta-analysis of the effectiveness of strategies to increase participation in cervical cancer screening. Br J Cancer 9. Article; Early Access. DOI: 10.1038/s41416-022-02094-w. PMC9977737 36517552

[14] Chan CK Aimagambetova G Ukybassova T , et al. Human papillomavirus infection and cervical cancer: epidemiology, screening, and vaccination-review of current perspectives. J Oncol Print 2019; 2019: 3257939. 10.1155/2019/3257939 PMC6811952 31687023

[15] Official statistics National statistics. Cervical screening programme 2017–18 [NS], https://digital.nhs.uk/data-and-information/publications/statistical/cervical-screening-annual/england—2017-18 (2018, accessed 4.5.2023).

[16] Chorley AJ Marlow LA Forster AS , et al. Experiences of cervical screening and barriers to participation in the context of an organised programme: a systematic review and thematic synthesis. Psychooncology 2017; 26: 161–172. 27072589 10.1002/pon.4126 PMC5324630

[17] Waller J Bartoszek M Marlow L , et al. Barriers to cervical cancer screening attendance in England: a population-based survey. J Med Screen 2009; 16: 199–204. Research Support, Non-U.S. Gov’t. 20054095 10.1258/jms.2009.009073

[18] Marlow LA Waller J Wardle J . Barriers to cervical cancer screening among ethnic minority women: a qualitative study. J Fam Plann Reprod Health Care 2015; 41: 248–254. Research Support, Non-U.S. Gov’t. 25583124 10.1136/jfprhc-2014-101082 PMC4621371

[19] Mackie A UHSA. Health matters: making cervical cancer screening more accessible, https://www.gov.uk/government/publications/health-matters-making-cervical-screening-more-accessible/health-matters-making-cervical-screening-more-accessible–2 (2017, accessed 24.10.2022).

[20] Berner AM Connolly DJ Pinnell I , et al. Attitudes of transgender men and non-binary people to cervical screening: a cross-sectional mixed-methods study in the UK. Br J Gen Pract 2021; 71: e614–e625. 10.3399/BJGP.2020.0905 PMC8136582 34001539

[21] Douglas E Waller J Duffy SW , et al. Socioeconomic inequalities in breast and cervical screening coverage in England: are we closing the gap? J Med Screen 2016; 23: 98–103. 26377810 10.1177/0969141315600192 PMC4855247

[22] Lancucki L Fender M Koukari A , et al. A fall-off in cervical screening coverage of younger women in developed countries. J Med Screen 2010; 17: 91–96. 20660438 10.1258/jms.2010.010017

[23] Marlow LAV Chorley AJ Haddrell J , et al. Understanding the heterogeneity of cervical cancer screening non-participants: data from a national sample of British women. Eur J Cancer 2017; 80: 30–38. Research Support, Non-U.S. Gov’t. 28535495 10.1016/j.ejca.2017.04.017 PMC5489076

[24] Reynolds F Stanistreet D Elton P . Women with learning disabilities and access to cervical screening: retrospective cohort study using case control methods. BMC Public Health 2008; 8: 30. 18218106 10.1186/1471-2458-8-30 PMC2248570

[25] Cancer Research UK. Cervical screening, https://www.cancerresearchuk.org/health-professional/screening/cervical-screening#Evidencecervical1 (accessed 15.10.2022).

[26] Nishimura H Yeh PT Oguntade H , et al. HPV self-sampling for cervical cancer screening: a systematic review of values and preferences. BMJ Global Health 2021; 6: 05. Research Support, Non-U.S. Gov’t Systematic Review. 10.1136/bmjgh-2020-003743 PMC8137189 34011537

[27] Snijders PJ Verhoef VM Arbyn M , et al. High-risk HPV testing on self-sampled versus clinician-collected specimens: a review on the clinical accuracy and impact on population attendance in cervical cancer screening. Int J Cancer 2013; 132: 2223–2236. Research Support, Non-U.S. Gov’t Review. 22907569 10.1002/ijc.27790

[28] Nelson EJ Maynard BR Loux T , et al. The acceptability of self-sampled screening for HPV DNA: a systematic review and meta-analysis. Sex Transm Infect 2017; 93: 56–61. 28100761 10.1136/sextrans-2016-052609

[29] Yeh PT Kennedy CE de Vuyst H , et al. Self-sampling for human papillomavirus (HPV) testing: a systematic review and meta-analysis. BMJ Global Health 2019; 4: e001351. 10.1136/bmjgh-2018-001351 PMC6529022 31179035

[30] Cadman L Wilkes S Mansour D , et al. A randomized controlled trial in non-responders from Newcastle upon Tyne invited to return a self-sample for human papillomavirus testing versus repeat invitation for cervical screening. J Med Screen 2015; 22: 28–37. 25403717 10.1177/0969141314558785

[31] Lozar T Nagvekar R Rohrer C , et al. Cervical cancer screening postpandemic: self-sampling opportunities to accelerate the elimination of cervical cancer. Int J Womens Health 2021; 13: 841–859. 34566436 10.2147/IJWH.S288376 PMC8458024

[32] Bonde J . Self-sampling to reach non-participating women, https://www.hpvworld.com/articles/self-sampling-to-reach-non-participating-women/ (accessed 30.10.2022).

[33] European Cancer Organisation. Self-sampling and HPV screening in Europe: Position Paper, https://www.europeancancer.org/policy/13-policy/27-self-sampling-and-hpv-screening-in-europe (2021, accessed 26.10.2022).

[34] Serrano B Ibanez R Robles C , et al. Worldwide use of HPV self-sampling for cervical cancer screening. Prev Med 2022; 154: 106900. Research Support, Non-U.S. Gov’t Systematic Review. 34861338 10.1016/j.ypmed.2021.106900

[35] Lim A Deats K Gambell J , et al. Opportunistic offering of self-sampling to non-attenders within the English cervical screening programme: a pragmatic, multicentre, implementation feasibility trial with randomly allocated cluster intervention start dates (Youscreen). *Available at SSRN* 2023. DOI: dx.doi.org/10.2139/ssrn.4555189. 10.1016/j.eclinm.2024.102672 PMC11490653 39429813

[36] Page MJ McKenzie JE Bossuyt PM , et al. The PRISMA 2020 statement: an updated guideline for reporting systematic reviews. Br Med J 2021; 372: n71. Research Support, N.I.H., Extramural Research Support, Non-U.S. Gov’t. 10.1136/bmj.n71 PMC8005924 33782057

[37] Jo’s cervical cancer trust. What is HPV self-sampling?, https://www.jostrust.org.uk/about-us/news-and-blog/blog/what-hpv-self-sampling (2021, accessed 5.3.2023).

[38] Popay J Roberts H Sowden A , et al. Guidance on the conduct of narrative synthesis in systematic reviews: a product of the ESRC methods programme. Lancaster, UK: University of Lancaster, 2006.

[39] Sterne JAC Savovic J Page MJ , et al. RoB 2: a revised tool for assessing risk of bias in randomised trials. The BMJ 2019; 1: l4898. 10.1136/bmj.l4898 31462531

[40] Sterne JA Hernan MA Reeves BC , et al. ROBINS-I: a tool for assessing risk of bias in non-randomised studies of interventions. Br Med J 2016; 355: i4919. 10.1136/bmj.i4919 PMC5062054 27733354

[41] Cochrane Scientific Committee. Review of the development of the risk of bias tool for nonrandomised studies for interventions – ROBINS-I, https://methods.cochrane.org/sites/methods.cochrane.org/files/uploads/scientific_committee_statement_report_robins_i_fin.pdf (2017, accessed 17.4.2023).

[42] Gök M Heideman DAM van Kemenade FJ , et al. Offering self-sampling for human papillomavirus testing to non-attendees of the cervical screening programme: characteristics of the responders. Eur J Cancer 2012; 48: 1799–1808. 22172570 10.1016/j.ejca.2011.11.022

[43] Gok M van Kemenade FJ Heideman DA , et al. Experience with high-risk human papillomavirus testing on vaginal brush-based self-samples of non-attendees of the cervical screening program. Int J Cancer 2012; 130: 1128–1135. Randomized Controlled Trial Research Support, Non-U.S. Gov’t. 21484793 10.1002/ijc.26128

[44] Gok M Heideman DA van Kemenade FJ , et al. HPV Testing on self collected cervicovaginal lavage specimens as screening method for women who do not attend cervical screening: cohort study. Br Med J 2010; 340: c1040. Controlled Clinical Trial Research Support, Non-U.S. Gov’t. 10.1136/bmj.c1040 PMC2837143 20223872

[45] Anhang R Nelson JA Telerant R , et al. Acceptability of self-collection of specimens for HPV DNA testing in an urban population. J Womens Health 2005; 14: 721–728. 10.1089/jwh.2005.14.721 16232104

[46] Kilfoyle KA Marais ACD Mai Anh N , et al. Preference for human papillomavirus self-collection and papanicolaou: survey of underscreened women in North Carolina. J Low Genit Tract Dis 2018; 22: 302–310. 30179994 10.1097/LGT.0000000000000430 PMC6174678

[47] Pretsch PK Spees LP Brewer NT , et al. Effect of HPV self-collection kits on cervical cancer screening uptake among under-screened women from low-income US backgrounds (MBMT-3): a phase 3, open-label, randomised controlled trial. Lancet Public Health 2023; 8: e411–e421. 10.1016/S2468-2667(23)00076-2 PMC10283467 37182529

[48] Carrasquillo O Seay J Amofah A , et al. HPV Self-Sampling for cervical cancer screening among ethnic minority women in south Florida: a randomized trial. JGIM: J Gen Intern Med 2018; 33: 1077–1083. 29594933 10.1007/s11606-018-4404-z PMC6025679

[49] Castle PE Rausa A Walls T , et al. Comparative community outreach to increase cervical cancer screening in the Mississippi Delta. Prev Med 2011; 52: 452–455. 21497619 10.1016/j.ypmed.2011.03.018 PMC3114876

[50] Kobetz E Seay J Koru-Sengul T , et al. A randomized trial of mailed HPV self-sampling for cervical cancer screening among ethnic minority women in South Florida. Cancer Causes Control 2018; 29: 793–801. 29995217 10.1007/s10552-018-1055-7 PMC6329676

[51] Molokwu JC Penaranda E Dwivedi A , et al. Effect of educational intervention on self-sampling acceptability and follow-up paps in border dwelling Hispanic females. J Low Genit Tract Dis 2018; 22: 295–301. 30138152 10.1097/LGT.0000000000000424

[52] Sewali B Okuyemi KS Askhir A , et al. Cervical cancer screening with clinic-based Pap test versus home HPV test among Somali immigrant women in Minnesota: a pilot randomized controlled trial. Cancer Med 2015; 4: 620–631. 25653188 10.1002/cam4.429 PMC4402076

[53] Waller J McCaffery K Forrest S , et al. Acceptability of unsupervised HPV self-sampling using written instructions. J Med Screen 2006; 13: 208–213. 17217611 10.1177/096914130601300409

[54] Landy R Hollingworth T Waller J , et al. Non-speculum sampling approaches for cervical screening in older women: randomised controlled trial. Br J Gen Pract 2022; 72: e26–e33. 10.3399/BJGP.2021.0350 PMC8714504 34972808

[55] Wedisinghe L Sasieni P Currie H , et al. The impact of offering multiple cervical screening options to women whose screening was overdue in Dumfries and Galloway, Scotland. Prev Med Rep 2022; 29. Article. DOI: 10.1016/j.pmedr.2022.101947. PMC9502330 36161116

[56] Kitchener H Gittins M Cruickshank M , et al. A cluster randomized trial of strategies to increase uptake amongst young women invited for their first cervical screen: the strategic trial. J Med Screen 2018; 25: 88–98. 28530513 10.1177/0969141317696518 PMC5956569

[57] Brewer N Bartholomew K Grant J , et al. Acceptability of human papillomavirus (HPV) self-sampling among never- and under-screened Indigenous and other minority women: a randomised three-arm community trial in Aotearoa, New Zealand. Lancet Reg Health West Pac 2021; 16. DOI: 10.1016/j.lanwpc.2021.100265. PMC8427317 34590066

[58] MacDonald EJ Geller S Sibanda N , et al. Reaching under-screened/never-screened indigenous peoples with human papilloma virus self-testing: a community-based cluster randomised controlled trial. Aust N Z J Obstet Gynaecol 2021; 61: 135–141. 33350455 10.1111/ajo.13285

[59] Virtanen A Anttila A Luostarinen T , et al. Self-sampling versus reminder letter: effects on cervical cancer screening attendance and coverage in Finland. Int J Cancer 2011; 128: 2681–2687. 20669228 10.1002/ijc.25581

[60] Virtanen A Anttila A Luostarinen T , et al. Improving cervical cancer screening attendance in Finland. Int J Cancer 2015; 136: E677–E684. 10.1002/ijc.29176 25178683

[61] Sultana F English DR Simpson JA , et al. Home-based HPV self-sampling improves participation by never-screened and under-screened women: results from a large randomized trial (iPap) in Australia. Int J Cancer 2016; 139: 281–290. 26850941 10.1002/ijc.30031

[62] Kellen E Benoy I Vanden Broeck D , et al. A randomized, controlled trial of two strategies of offering the home-based HPV self-sampling test to non- participants in the Flemish cervical cancer screening program. Int J Cancer 2018; 143: 861–868. 29569715 10.1002/ijc.31391

[63] Tranberg M Bech BH Blaakær J , et al. HPV self-sampling in cervical cancer screening: the effect of different invitation strategies in various socioeconomic groups – a randomized controlled trial. Clin Epidemiol 2018; 10: 1027–1036. 30197540 10.2147/CLEP.S164826 PMC6112594

[64] Sancho-Garnier H Tamalet C Halfon P , et al. HPV self-sampling or the Pap-smear: a randomized study among cervical screening nonattenders from lower socioeconomic groups in France. Int J Cancer 2013; 133: 2681–2687. 23712523 10.1002/ijc.28283

[65] Ngu SF Lau LSK Li JS , et al. Human papillomavirus self-sampling for primary cervical cancer screening in under-screened women in Hong Kong during the COVID-19 pandemic. Int J Environ Res Public Health 2022; 19: 11. 10.3390/ijerph19052610 PMC8910259 35270303

[66] Anand KV Mishra GA Pimple SA , et al. Cross-sectional study of HPV self-sampling among Indian women – a way forward. Indian J Med Paediatr Oncol 2022; 43: 103–108.

[67] McFarlane SJ Morgan SE Carcioppolo N . Lessons learned from the ‘Goodie Box’: A message design study developed and evaluated in community settings for cervical cancer prevention. Front Oncol 2022; 12. DOI: 10.3389/fonc.2022.935704. PMC9492837 36158662

[68] Lazcano-Ponce E Lorincz AT Cruz-Valdez A , et al. Self-collection of vaginal specimens for human papillomavirus testing in cervical cancer prevention (MARCH): a community-based randomised controlled trial. Lancet 2011; 378: 1868–1873. 22051739 10.1016/S0140-6736(11)61522-5

[69] Verdoodt F Jentschke M Hillemanns P , et al. Reaching women who do not participate in the regular cervical cancer screening programme by offering self-sampling kits: a systematic review and meta-analysis of randomised trials. Eur J Cancer 2015; 51: 2375–2385. 26296294 10.1016/j.ejca.2015.07.006

[70] Amir SM Idris IB Mohd Yusoff H . The acceptance of human papillomavirus self-sampling test among Muslim women: a systematic review. Asian Pac J Cancer Prev 2022; 23: 767–774. 35345345 10.31557/APJCP.2022.23.3.767 PMC9360951

[71] National Institute for Health and Care Research (NIHR). Improving inclusion of under-served groups in clinical research: Guidance from INCLUDE project, https://www.nihr.ac.uk/documents/improving-inclusion-of-under-served-groups-in-clinical-research-guidance-from-include-project/25435 (2020, accessed 17.4.2023).

