# Supplementary material for: Identifying optimum implementation for human papillomavirus self-sampling in underserved communities: A systematic review
Source: J Med Screen. 2024 Aug 30;32(1):2–18. doi: 10.1177/09691413241274312 (PMC11869506; doi:10.1177/09691413241274312)
Supplement: sj-docx-1-msc-10.1177_09691413241274312 - Supplemental material for Identifying optimum implementation for human papillomavirus self-sampling in underserved communities: A systematic review [file sj-docx-1-msc-10.1177_09691413241274312.docx]

Appendix A: Search strategy for each database

Medline , PsycInfo and EMBASE via Ovid

(HPV OR Human papillomavirus* OR Human papilloma virus* OR hrHPV OR cervix •OR cervical*)) AND (Self-screen*OR Self-screen* OR Self sampl* OR Self-sampl* OR self test* OR self administer* OR Self collect* OR Home test* OR Home based test* OR Home Kit*OR Home screen* )) AND (Non-attend* OR Non attend* OR nonattend* OR Non responder* OR Non-responder* OR Nonresponder* OR nonparticipant* OR Non-participant* OR Non participant* OR underserved OR LGBT* OR "Homosexual*" OR transgender OR Bisexual* OR gay OR queer OR lesbian* OR transsexual* OR Socioeconomic status* OR Socio-economic status* OR Socio economic status* OR Social class* OR Social status* OR Deprived* OR Deprivation* OR Low-income* OR young* OR Young* women OR Young* woman OR Old* women OR Old* woman OR Old OR post menopaus* OR post-menopause* OR religi* OR cultur* OR Ethnic minorit* OR BAME OR Black Asian and minority ethnic OR black OR asian* OR Minority group* OR emigrant* OR immigrant* OR Learning disabilit* OR Physical-disabilit* OR disabled )

Scopus

( TITLE-ABS-KEY ( hpv  OR  "Human papillomavirus*"  OR  "Human papillomavirus* "  OR  hrhpv  OR  cervix  OR  cervical* ) )  AND  ( TITLE-ABS-KEY ( "Self screen*"  OR  "Self-screen*"  OR  "Self sampl*"  OR  " Self-sampl*"  OR  "Self test*"  OR  "Self administer*"  OR  "Self collect*"  OR  "Home test*"  OR  "Home based test*"  OR  "Home Kit*"  OR  "Home screen*" ) )  AND  ( TITLE-ABS-KEY ( "Non-attend*"  OR  "Non attend*"  OR  nonattend*  OR  "Non responder*"  OR  "Non-responder*"  OR  "Nonresponder*"  OR  nonparticipant*  OR  "Non-participant*"  OR  "Non participant*"  OR  underserved  OR  "LGBT*"  OR  "Homosexual*"  OR  transgender  OR  "Bisexual*"  OR  gay  OR  queer  OR  lesbian*  OR  transsexual*  OR  "Socioeconomic status*"  OR  "Socio-economic status*"  OR  "Socio economic status*"  OR  "Social class*"  OR  "Social status*"  OR  "Deprived*"  OR  "Deprivation*"  OR  "Low-income*"  OR  young*  OR  "Young* women"  OR  "Young* woman"  OR  "Old* women"  OR  "Old* woman"  OR  "Old"  OR  "post menopaus*"  OR  "post-menopause*"  OR  religi*  OR  cultur*  OR  "Ethnic minorit*"  OR  "BAME"  OR  "Black Asian and minority ethnic"  OR  black  OR  asian*  OR  "Minority group*"  OR  emigrant*  OR  immigrant*  OR  "Learning disabilit*"  OR  "Physical-disabilit*"  OR  disabled ) )

Web of science:

((TS=(HPV OR "Human papillomavirus*" OR "Human papilloma virus*" OR hrHPV OR cervix •OR "cervical*")) AND TS=("Self screen*" OR" Self-screen*" OR "Self sampl*" OR "Self-sampl*" OR "Self test*" OR "Self administer*" OR "Self collect*"OR"Home test*" OR "Home based test*" OR "Home Kit*"OR "Home screen*" )) AND TS=("Non-attend*" OR "Non attend*" OR nonattend* OR "Non responder*" OR "Non-responder*" OR "Nonresponder*" OR nonparticipant* OR "Non-participant*" OR "Non participant*" OR underserved OR "LGBT*" OR "Homosexual*" OR transgender OR "Bisexual*" OR gay OR queer OR lesbian* OR transsexual* OR "Socioeconomic status*" OR "Socio-economic status*" OR "Socio economic status*" OR "Social class*" OR "Social status*" OR "Deprived*" OR "Deprivation*" OR "Low-income*" OR young* OR "Young* women" OR "Young* woman" OR "Old* women" OR "Old* woman" OR "Old" OR "post menopaus*" OR "post-menopause*" OR religi* OR cultur* OR "Ethnic minorit*" OR "BAME" OR "Black Asian and minority ethnic" OR black OR asian* OR "Minority group*" OR emigrant* OR immigrant* OR "Learning disabilit*" OR "Physical-disabilit*" OR disabled )

CINAHL:

(HPV OR "Human papillomavirus*" OR "Human papilloma virus*" OR hrHPV OR cervix •OR "cervical*")) AND ("Self-screen*" OR" Self-screen*" OR "Self sampl*" OR "Self-sampl*" OR "Self test*" OR "Self administer*" OR "Self collect*"OR"Home test*" OR "Home based test*" OR "Home Kit*"OR "Home screen*" )) AND ("Non-attend*" OR "Non attend*" OR nonattend* OR "Non responder*" OR "Non-responder*" OR "Nonresponder*" OR nonparticipant* OR "Non-participant*" OR "Non participant*" OR underserved OR "LGBT*" OR "Homosexual*" OR transgender OR "Bisexual*" OR gay OR queer OR lesbian* OR transsexual* OR "Socioeconomic status*" OR "Socio-economic status*" OR "Socio economic status*" OR "Social class*" OR "Social status*" OR "Deprived*" OR "Deprivation*" OR "Low-income*" OR young* OR "Young* women" OR "Young* woman" OR "Old* women" OR "Old* woman" OR "Old" OR "post menopaus*" OR "post-menopause*" OR religi* OR cultur* OR "Ethnic minorit*" OR "BAME" OR "Black Asian and minority ethnic" OR black OR asian* OR "Minority group*" OR emigrant* OR immigrant* OR "Learning disabilit*" OR "Physical-disabilit*" OR disabled )
