# Supplementary material for: Identifying optimum implementation for human papillomavirus self-sampling in underserved communities: A systematic review
Source: J Med Screen. 2024 Aug 30;32(1):2–18. doi: 10.1177/09691413241274312 (PMC11869506; doi:10.1177/09691413241274312)
Supplement: sj-docx-2-msc-10.1177_09691413241274312 - Supplemental material for Identifying optimum implementation for human papillomavirus self-sampling in underserved communities: A systematic review [file sj-docx-2-msc-10.1177_09691413241274312.docx]

Appendix B: Data Extraction Form

| **Study (Country)** | **Country** | **Study design and aim** | **Participants & setting** | **Implementation strategies compared** | **Outcomes** | **Results** | **Observations/notes** |
| --- | --- | --- | --- | --- | --- | --- | --- |
| **Autor, year**  **(Country)** |  | **Study Design:**  **Aim:** | **Sample size:**  **Participants:**  **Setting:**  **Dates of data collection:** |  |  |  |  |
