# Supplementary material for: Identifying optimum implementation for human papillomavirus self-sampling in underserved communities: A systematic review
Source: J Med Screen. 2024 Aug 30;32(1):2–18. doi: 10.1177/09691413241274312 (PMC11869506; doi:10.1177/09691413241274312)
Supplement: sj-docx-3-msc-10.1177_09691413241274312 - Supplemental material for Identifying optimum implementation for human papillomavirus self-sampling in underserved communities: A systematic review [file sj-docx-3-msc-10.1177_09691413241274312.docx]

**Appendix C: Papers excluded at full-text level**

| Paper | Reason |
| --- | --- |
| Adcock A, Lawton B, MacDonald EJ, Hart S, Cram F, Geller S, et al. Acceptability of self‐taken vaginal HPV sample for cervical screening among an under‐screened Indigenous population. Australian & New Zealand Journal of Obstetrics & Gynaecology. 2019;59(2):301-7. doi: 10.1111/ajo.12933 | No comparator group |
| Adegboyega A, Wiggins AT, Williams LB, Dignan M. HPV Testing Behaviors and Willingness to Use HPV Self-sampling at Home Among African American (AA) and Sub-Saharan African Immigrant (SAI) Women. J Racial Ethn Health Disparities. 2022;9(6):2485-94. doi: 10.1007/s40615-021-01184-4 | Hypothetical |
| Afzal O, Lieb W, Lieber M, Chowdhury S, Beddoe AM. Cervical cancer risk factors and screening preferences among muslim women in Monrovia, Liberia. African Journal of Reproductive Health. 2020;24(3):101-7. doi: 10.29063/ajrh2020/v24i3.11 | No comparator group |
| Anderson C, Breithaupt L, Des Marais A, Rastas C, Richman A, Barclay L, et al. Acceptability and ease of use of mailed HPV self-collection among infrequently screened women in North Carolina. Sexually Transmitted Infections. 2018;94(2):131-7. doi: 10.1136/sextrans-2017-053235 | No comparator group |
| Arrossi S. Introduction of HPV self-collection in Argentina, main results, and lessons. Cancer Epidemiology Biomarkers and Prevention Conference: 12th AACR Conference on the Science of Cancer Health Disparities in Racial/Ethnic Minorities and the Medically Underserved San Francisco, CA United States. 2020;29(6 SUPPL 2) doi: https://dx.doi.org/10.1158/1538-7755.DISP19-IA13 | Not full paper |
| Awua AK, Wiredu EK, Afari EA, Tijani AS, Djanmah G, Adanu RMK. A tailored within-community specimen collection strategy increased uptake of cervical cancer screening in a cross-sectional study in Ghana. BMC Public Health. 2017;18(1) doi: 10.1186/s12889-017-4631-y | Not underserved population |
| Barbee L, Kobetz E, Menard J, Cook N, Blanco J, Barton B, et al. Assessing the acceptability of self-sampling for HPV among haitian immigrant women: CBPR in action. CANCER CAUSES CONTROL. 2010;21(3):421-31. doi: 10.1007/s10552-009-9474-0 | No comparator group |
| Bartholomew K. HPV self-sampling-all or not? Australian and New Zealand Journal of Obstetrics and Gynaecology. 2017;57(Supplement 1):9. doi: https://dx.doi.org/10.1111/ajo.12723 | Not full paper |
| Bertucci M, Bonnet E, Satger L, Kreiche A, Chappert JL, Loy-Morel S, et al. Acceptability of vaginal self-sampling with high-risk human papillomavirus testing for cervical cancer screening: a French questionnaire-based study. Women & health. 2021;61(1):83-94. doi: https://dx.doi.org/10.1080/03630242.2020.1831683 | No comparator group |
| Biederman E, Champion V, Zimet G. A conjoint analysis study on self-sampling for human papillomavirus (HPV) testing characteristics among black women in Indiana. BMC Women's Health. 2020;20(1) doi: 10.1186/s12905-020-00921-x | Hypothetical |
| Biederman EB. Factors associated with home based self-collection for human papillomavirus (HPV) testing. Dissertation Abstracts International: Section B: The Sciences and Engineering. 2022;83(5-B):No Pagination Specified. | Hypothetical |
| Brandt T, Wubneh SB, Handebo S, Debalkie G, Ayanaw Y, Alemu K, et al. Genital self-sampling for HPV-based cervical cancer screening: A qualitative study of preferences and barriers in rural Ethiopia. BMC Public Health. 2019;19(1) doi: 10.1186/s12889-019-7354-4 | Not underserved population |
| Brewer N, Foliaki S, Bromhead C, Viliamu-Amusia I, Pelefoti-Gibson L, Jones T, et al. Acceptability of human papillomavirus self-sampling for cervical-cancer screening in under-screened māori and pasifika women: A pilot study. New Zealand Medical Journal. 2019;132(1497):21-31. | Outcome of interest not reported |
| Broquet C, Triboullier D, Untiet S, Schafer S, Petignat P, Vassilakos P. Acceptability of self-collected vaginal samples for HPV testing in an urban and rural population of Madagascar. Afr Health Sci. 2015;15(3):755-61. doi: 10.4314/ahs.v15i3.8 | Not underserved population |
| Campbell HE, Gray AM, Watson J, Jackson C, Moseley C, Cruickshank ME, et al. Preferences for interventions designed to increase cervical screening uptake in non-attending young women: How findings from a discrete choice experiment compare with observed behaviours in a trial. Health Expect. 2020;23(1):202-11. doi: 10.1111/hex.12992 | Hypothetical |
| Carrasquillo O, Kobetz-Kerman EN, Alonzo Y. A RANDOMIZED TRIAL OF SELF-SAMPLING FOR HUMAN PAPILLOMA VIRUS AMONG MINORITY IMMIGRANT WOMEN IN NEED OF CERVICAL CANCER SCREENING: FINDINGS FROM THE SOUTH FLORIDA CENTER FOR REDUCING CANCER DISPARITIES. Journal of General Internal Medicine. 2015;30:S90-S. | Not full paper |
| Carrasquillo O, Rodriguez B, Kobetz-Kerman EN. A randomized trial ofa community health worker led intervention using hpv self-sampling to increase cervical cancer screening among minority women: Preliminary findings. Journal of General Internal Medicine. 2013;1):S12-S3. | Not full paper |
| Catarino R, Vassilakos P, Stadali-Ullrich H, Royannez-Drevardl I, Guillot C, Petignat P. Feasibility of At-Home Self-Sampling for HPV Testing as an Appropriate Screening Strategy for Nonparticipants in Switzerland: Preliminary Results of the DEPIST Study. Journal of Lower Genital Tract Disease. 2015;19(1):27-34. | No comparator group |
| Cerigo H, Coutlée F, Franco EL, Brassard P. Dry self-sampling versus provider-sampling of cervicovaginal specimens for human papillomavirus detection in the Inuit population of Nunavik, Quebec. Journal of Medical Screening. 2012;19(1):42-8. doi: 10.1258/jms.2012.012011 | Outcome of interest not reported |
| Darlin L, Borgfeldt C, Forslund O, Hénic E, Hortlund M, Dillner J, et al. Comparison of use of vaginal HPV self-sampling and offering flexible appointments as strategies to reach long-term non-attending women in organized cervical screening. Journal of Clinical Virology. 2013;58(1):155-60. doi: 10.1016/j.jcv.2013.06.029 | Not underserved population |
| De Alba I, Anton-Culver H, Hubbell FA, Ziogas A, Hess JR, Bracho A, et al. Self-sampling for human papillomavirus in a community setting: Feasibility in Hispanic women. Cancer Epidemiol Biomarkers Prev. 2008;17(8):2163-8. doi: 10.1158/1055-9965.EPI-07-2935 | No comparator group |
| Del Mistro A, Frayle H, Ferro A, Fantin G, Altobelli E, Giorgi Rossi P. Efficacy of self-sampling in promoting participation to cervical cancer screening also in subsequent round. Preventive Medicine Reports. 2017;5:166-8. doi: 10.1016/j.pmedr.2016.12.017 | Not underserved population |
| Des Marais AC, Brewer NT, Knight S, Smith JS. Patient perspectives on cervical cancer screening interventions among underscreened women. PLoS ONE. 2022;17(12 December) doi: 10.1371/journal.pone.0277791 | Hypothetical |
| Elfstrom KM, Sundstrom K, Andersson S, Bzhalava Z, Thor AC, Gzoul Z, et al. Increasing participation in cervical screening by targeting long-term nonattenders: Randomized health services study. International Journal of Cancer. 2019;145(11):3033-9. doi: 10.1002/ijc.32374 | Not underserved population |
| Fujita M, Nagashima K, Shimazu M, Suzuki M, Tauchi I, Sakuma M, et al. Implementation of a self-sampling HPV test for non-responders to cervical cancer screening in Japan: secondary analysis of the ACCESS trial. Scientific Reports. 2022;12(1) doi: 10.1038/s41598-022-18800-w | Not underserved population |
| Galbraith KV, Gilkey MB, Smith JS, Richman AR, Barclay L, Brewer NT. Perceptions of Mailed HPV Self-testing Among Women at Higher Risk for Cervical Cancer. Journal of Community Health. 2014;39(5):849-56. doi: 10.1007/s10900-014-9931-x | No comparator group |
| Gamelin R, Hébert M, Tratt E, Brassard P. Ethnographic study of the barriers and facilitators to implementing human papillomavirus (HPV) self-sampling as a primary screening strategy for cervical cancer among Inuit women of Nunavik, Northern Quebec. Int J Circumpolar Health. 2022;81(1) doi: 10.1080/22423982.2022.2032930 | No comparator group |
| Gizaw M, Ruddies F, Addissie A, Worku A, Abebe T, Teka B, et al. Community-based uptake of self-sampling for HPV DNA-based testing for cervical cancer screeningin Ethiopia: Preliminary findings of a cluster randomized trial. Cancer Epidemiology Biomarkers and Prevention Conference: 11th AACR Conference on the Science of Cancer Health Disparities in Racial/Ethnic Minorities and the Medically Underserved New Orleans, LA United States. 2020;29(6 SUPPL 1) doi: https://dx.doi.org/10.1158/1538-7755.DISP18-B103 | Not full paper |
| Goldstein Z, Martinson T, Ramachandran S, Lindner R, Safer JD. Improved Rates of Cervical Cancer Screening among Transmasculine Patients through Self-Collected Swabs for High-Risk Human Papillomavirus DNA Testing. Transgender Health. 2020;5(1):10-7. doi: 10.1089/trgh.2019.0019 | No comparator group |
| Hallik R, Innos K, Jänes J, Veerus P. HPV self-sampling among cervical cancer screening nonattenders: a feasibility study in Estonia...14th European Public Health Conference (Virtual), Public health futures in a changing world, November 10-12, 2021. European Journal of Public Health. 2021;31:iii138-iii9. | Not full paper |
| Huchko MJ, Olwanda E, Choi Y, Kahn JG. HPV-based cervical cancer screening in low-resource settings: Maximizing the efficiency of community-based strategies in rural Kenya. International Journal of Gynecology and Obstetrics. 2020;148(3):386-91. doi: 10.1002/ijgo.13090 | Not underserved population |
| Ilangovan K, Kobetz-Kerman EN, Koru-Sengul T, Marcus EN, Rodriguez B, Alonzo Y, et al. Acceptability and feasibility of HPV self-sampling for cervical cancer screening among patients and providers in two safety-net institutions in Miami. Journal of General Internal Medicine. 2014;1):S15-S6. | No comparator group |
| Ivanus U, Jerman T, Fokter AR, Takac I, Prevodnik VK, Marcec M, et al. Randomised trial of HPV self-sampling among non-attenders in the Slovenian cervical screening programme ZORA: Comparing three different screening approaches. Radiol Oncol. 2018;52(4):399-412. doi: 10.2478/raon-2018-0036 | Not underserved population |
| Jalili F, O’Conaill C, Templeton K, Lotocki R, Fischer G, Manning L, et al. Assessing the impact of mailing self-sampling kits for human papillomavirus testing to unscreened non-responder women in Manitoba. Current Oncology. 2019;26(3):167-72. doi: 10.3747/co.26.4575 | Not underserved population |
| Katz ML, Zimmermann BJ, Moore D, Paskett ED, Reiter PL. Perspectives from health-care providers and women about completing human papillomavirus (HPV) self-testing at home. Women & Health. 2017;57(10):1161-77. doi: 10.1080/03630242.2016.1243608 | No comparator group |
| Khoo SP, Lim WT, Rajasuriar R, Nasir NH, Gravitt P, Woo YL. The acceptability and preference of vaginal self-sampling for Human Papillomavirus (HPV) testing among a multi-ethnic asian female population. Cancer Prev Res. 2021;14(1):105-11. doi: 10.1158/1940-6207.CAPR-20-0280 | No comparator group |
| Lilliecreutz C, Karlsson H, Holm ACS. Participation in interventions and recommended follow-up for non-attendees in cervical cancer screening -taking the women’s own preferred test method into account—A Swedish randomised controlled trial. PLoS ONE. 2020;15(7) doi: 10.1371/journal.pone.0235202 | Outcome of interest not reported |
| Lim AWW, Hollingworth A, Kalwij S, Curran G, Sasieni P. Offering self-sampling to cervical screening non-attenders in primary care. Journal of Medical Screening. 2017;24(1):43-9. doi: 10.1177/0969141316639346 | Not underserved population |
| Lofters AK, Vahabi M, Fardad M, Raza A. Exploring the acceptability of human papillomavirus self-sampling among Muslim immigrant women. Cancer Manage Res. 2017;9:323-9. doi: 10.2147/CMAR.S139945 | Hypothetical |
| Lorenzi NPC, Termini L, Longatto Filho A, Tacla M, de Aguiar LM, Beldi MC, et al. Age-related acceptability of vaginal self-sampling in cervical cancer screening at two university hospitals: a pilot cross-sectional study. BMC Public Health. 2019;19(1):963. doi: 10.1186/s12889-019-7292-1 | No comparator group |
| Ma'som M, Bhoo-Pathy N, Nasir NH, Bellinson J, Subramaniam S, Ma Y, et al. Attitudes and factors affecting acceptability of self-administered cervicovaginal sampling for human papillomavirus (HPV) genotyping as an alternative to Pap testing among multiethnic Malaysian women. BMJ Open. 2016;6(8) (no pagination) doi: https://dx.doi.org/10.1136/bmjopen-2015-011022 | No comparator group |
| Maza M, Melendez M, Masch R, Alonzo T, Castle P, Soler M, et al. Hpv self-sampling in non-attenders of cervical cancer screening programs in El Salvador. International Journal of Gynecology and Obstetrics. 2018;143(Supplement 3):441. doi: https://dx.doi.org/10.1002/ijgo.12582 | Not underserved population |
| Mbatha JN, Galappaththi-Arachchige HN, Mtshali A, Taylor M, Ndhlovu PD, Kjetland EF, et al. Self-sampling for human papillomavirus testing among rural young women of KwaZulu-Natal, South Africa. BMC research notes. 2017;10(1):702. doi: https://dx.doi.org/10.1186/s13104-017-3045-3 | Not underserved population |
| McDowell M, Pardee DJ, Peitzmeier S, Reisner SL, Agénor M, Alizaga N, et al. Cervical cancer screening preferences among trans-masculine individuals: Patient-collected human papillomavirus vaginal swabs versus provider-administered pap tests. LGBT Health. 2017;4(4):252-9. doi: 10.1089/lgbt.2016.0187 | Hypothetical |
| McFarlane SJ, Morgan SE, Schlumbrecht M. Acceptability of a multicomponent, community-based, HPV self-test intervention among Jamaican women. Cancer Causes & Control. 2021;32(5):547-54. doi: 10.1007/s10552-021-01406-4 | No comparator group |
| Miller EM. Evaluating implementation of HPV self-collection for cervical cancer screening among low-income, under-screened women in the US. Pharmacoepidemiol Drug Saf. 2022;31:468-. | Not full paper |
| Mitchell EM, Lothamer H, Garcia C, Forera M, Al Kallas H, Pokam Tchuisseu Y, et al. Exploring access to cervical cancer screening through at-home self-collection and hpv testing: Lessons learned in the two rural resource-limited settings of southwest virginia, usa and Bluefields, Nicaragua. Annals of Global Health. 2017;83(1):195. | Not full paper |
| Mitchell EM, Lothamer H, Garcia C, Marais AD, Camacho F, Poulter M, et al. Acceptability and Feasibility of Community-Based, Lay Navigator-Facilitated At-Home Self-Collection for Human Papillomavirus Testing in Underscreened Women. Journal of Women's Health. 2020;29(4):596-602. doi: 10.1089/jwh.2018.7575 | No comparator group |
| Montealegre JR, Anderson ML, Hilsenbeck SG, Chiao EY, Cantor SB, Parker SL, et al. Mailed self-sample HPV testing kits to improve cervical cancer screening in a safety net health system: protocol for a hybrid effectiveness-implementation randomized controlled trial. Trials. 2020;21(1) doi: 10.1186/s13063-020-04790-5 | Not full paper |
| Mremi A, Linde DS, McHome B, Mlay J, Schledermann D, Blaakær J, et al. Acceptability and feasibility of self-sampling and follow-up attendance after text message delivery of human papillomavirus results: A cross-sectional study nested in a cohort in rural Tanzania. Acta Obstetricia et Gynecologica Scandinavica. 2021;100(4):802-10. doi: 10.1111/aogs.14117 | No comparator group |
| Nakisige C, Trawin J, Mitchell-Foster S, Payne BA, Rawat A, Mithani N, et al. Integrated cervical cancer screening in Mayuge District Uganda (ASPIRE Mayuge): A pragmatic sequential cluster randomized trial protocol. BMC Public Health. 2020;20(1) doi: 10.1186/s12889-020-8216-9 | Not full paper |
| Oketch SY, Kwena Z, Choi Y, Adewumi K, Moghadassi M, Bukusi EA, et al. Perspectives of women participating in a cervical cancer screening campaign with community-based HPV self-sampling in rural western Kenya: a qualitative study. BMC Womens Health. 2019;19:10. doi: 10.1186/s12905-019-0778-2 | Not underserved population |
| Penaranda E, Molokwu J, Flores S, Byrd T, Shokar N. The effects of an educational intervention on self-sampling for human papillomavirus acceptability: RCT. Journal of Lower Genital Tract Disease. 2016;20(2 Supplement 1):S5. | Not full paper |
| Plafker B, Stamper S, Goldstein L, Lipson R, Bedell S, Wang J, et al. Patient Satisfaction with Human Papillomavirus Self-Sampling in a Cohort of Ethnically Diverse and Rural Women in Yunnan Province, China. Journal of Lower Genital Tract Disease. 2020;24(4):349-52. doi: https://dx.doi.org/10.1097/LGT.0000000000000560 | No comparator group |
| Rossi PG, Marsili LM, Camilloni L, Iossa A, Lattanzi A, Sani C, et al. The effect of self-sampled HPV testing on participation to cervical cancer screening in Italy: a randomised controlled trial (ISRCTN96071600). British Journal of Cancer. 2011;104(2):248-54. doi: 10.1038/sj.bjc.6606040 | Not underserved population |
| Sahlgren H, Sparén P, Elfgren K, Miriam Elfström K. Feasibility of sending a direct send HPV self-sampling kit to long-term non-attenders in an organized cervical screening program. European Journal of Obstetrics and Gynecology and Reproductive Biology. 2022;268:68-73. doi: 10.1016/j.ejogrb.2021.11.430 | Not underserved population |
| Santella C, Tratt E, Nyamiaka J, Whiteley Tukkiapik L, Styffe C, Gamelin R, et al. Perceptions of Inuit Women and Non-Inuit Healthcare Providers on the Implementation of Human Papillomavirus Self-Sampling as an Alternative Cervical Cancer Screening Method in Nunavik, Northern Quebec. Qual Health Res. 2022;32(8-9):1259-72. doi: 10.1177/10497323221090805 | Hypothetical |
| Scarinci I. An innovative community-participatory approach to address cervical cancer disparities among African American women in the rural Mississippi Delta. Psycho-Oncology. 2020;29(Supplement 1):16-7. doi: https://dx.doi.org/10.1002/pon.5327 | Not full paper |
| Schee K, Stefan L, Pedersen H, Bonde J, Nygard M. Improving attendance to the cervical cancer screening program: Does self-sampling at home improve cervical cancer prevention. Cancer Research Conference: 105th Annual Meeting of the American Association for Cancer Research, AACR. 2014;74(19 SUPPL. 1) doi: https://dx.doi.org/10.1158/1538-7445.AM2014-4135 | Not full paper |
| Sewali BP, Askhir A, Belinson J, Vogel RI, Okuyemi KS, Joseph A, et al. Clinic-based pap test versus HPV home test among somali immigrant women in Minnesota: A randomized controlled trail. Cancer Epidemiology Biomarkers and Prevention Conference: 7th AACR Conference on the Science of Health Disparities in Racial/Ethnic Minorities and the Medically Underserved San Antonio, TX United States Conference Publication:. 2015;24(10 SUPPL. 1) doi: https://dx.doi.org/10.1158/1538-7755.DISP14-PR03 | Not full paper |
| Sherman SM, Brewer N, Bartholomew K, Bromhead C, Crengle S, Cunningham C, et al. Human papillomavirus self-testing among unscreened and under-screened Māori, Pasifika and Asian women in Aotearoa New Zealand: A preference survey among responders and interviews with clinical-trial nonresponders. Health Expect. 2022;25(6):2914-23. doi: 10.1111/hex.13599 | No comparator group |
| Smith JS, Des Marais AC, Deal AM, Richman AR, Perez-Heydrich C, Yen-Lieberman B, et al. Mailed Human Papillomavirus Self-Collection with Papanicolaou Test Referral for Infrequently Screened Women in the United States. Sexually Transmitted Diseases. 2018;45(1):42-8. doi: 10.1097/OLQ.0000000000000681 | No comparator |
| Sultana F, Mullins R, Murphy M, English DR, Simpson JA, Drennan KT, et al. Women's views on human papillomavirus self-sampling: focus groups to assess acceptability, invitation letters and a test kit in the Australian setting. Sexual Health. 2015;12(4):279-86. doi: 10.1071/sh14236 | Hypothetical |
| Szarewski A, Cadman L, Ashdown-Barr L, Waller J. Exploring the acceptability of two self-sampling devices for human papillomavirus testing in the cervical screening context: A qualitative study of Muslim women in London. Journal of Medical Screening. 2009;16(4):193-8. doi: 10.1258/jms.2009.009069 | Hypothetical |
| Tamalet C, Halfon P, Retraite LL, Grob A, Leandri FX, Heid P, et al. Genotyping and follow-up of HR-HPV types detected by self-sampling in women from low socioeconomic groups not participating in regular cervical cancer screening in France. Journal of Clinical Virology. 2016;78:102-7. doi: 10.1016/j.jcv.2016.02.027 | Outcome of interest not reported |
| Tatari CR, Andersen B, Brogaard T, Badre-Esfahani S, Jaafar N, Kirkegaard P. The SWIM study: Ethnic minority women's ideas and preferences for a tailored intervention to promote national cancer screening programmes-A qualitative interview study. Health Expect. 2021;24(5):1692-700. doi: 10.1111/hex.13309 | Outcome of interest not reported |
| Vahabi M, Lofters A. Muslim immigrant women's views on cervical cancer screening and HPV self-sampling in Ontario, Canada. BMC Public Health. 2016;16(1) doi: 10.1186/s12889-016-3564-1 | Hypothetical |
| Veerus P, Hallik R, Janes J, Joers K, Paapsi K, Laidra K, et al. HPV self-sampling in cervical cancer screening: A randomised feasibility study in Estonia in 2020. Eesti Arst. 2022;101(Supplement 4):26. | Not full paper |
| Virtanen A, Nieminen P, Niironen M, Luostarinen T, Anttila A. Self-sampling experiences among non-attendees to cervical screening. Gynecologic Oncology. 2014;135(3):487-94. doi: 10.1016/j.ygyno.2014.09.019 | No comparator group |
| Winer RL, Lin J, Tiro JA, Miglioretti DL, Beatty T, Gao HY, et al. Effect of Mailed Human Papillomavirus Test Kits vs Usual Care Reminders on Cervical Cancer Screening Uptake, Precancer Detection, and Treatment: A Randomized Clinical Trial. Obstetrical & Gynecological Survey. 2020;75(3):167-8. doi: 10.1097/01.ogx.0000655424.57924.6b | Not underserved population |
| Wong ELY, Cheung AWL, Wong AYK, Chan PKS. Acceptability and feasibility of hpv self-sampling as an alternative primary cervical cancer screening in under-screened population groups: A cross-sectional study. International Journal of Environmental Research and Public Health. 2020;17(17):1-15. doi: 10.3390/ijerph17176245 | No comparator group |
